# Identification, isolation, and structural characterization of novel forced degradation products of Ertugliflozin using advanced analytical techniques

**DOI:** 10.1038/s41598-023-36289-9

**Published:** 2023-06-10

**Authors:** Suresh Salakolusu, Naresh Kumar Katari, Ganapavarapu Veera Raghava Sharma, Muralidharan Kaliyaperumal, Umamaheshwar Puppala, Mahesh Ranga, Sreekantha Babu Jonnalagadda

**Affiliations:** 1 Analytical Discovery Chemistry, Aragen Life Sciences Pvt. Ltd., IDA Nacharam, Hyderabad, 500076 India; 2 Department of Chemistry, GITAM School of Science, GITAM Deemed to be University, Visakhapatnam, 530045 Andhra Pradesh India; 3 Department of Chemistry, GITAM School of Science, GITAM Deemed to be University, Hyderabad, 502329 Telangana India; 4 School of Chemistry and Physics, College of Agriculture, Engineering and Science, Westville Campus, University of KwaZulu-Natal, P Bag X 54001, Durban, 4000 South Africa

**Keywords:** Analytical chemistry, Infrared spectroscopy, Mass spectrometry, NMR spectroscopy

## Abstract

The research elucidates the stress degradation behavior of Ertugliflozin, which is used for the treatment of type-2 diabetics. The degradation was conducted as per ICH guidelines and Ertugliflozin is relatively stable in thermal, photolytic, neutral, and alkaline hydrolysis conditions; however, considerable degradation was detected in acid hydrolysis and oxidative hydrolysis. Degradation products were identified by ultra-high-performance liquid chromatography-mass spectrometry, isolated by semi-preparative high-performance liquid chromatography, and structural characterization using high-resolution mass spectrometry and nuclear magnetic resonance spectroscopy. Total four degradation products were identified and isolated in acid degradation, which are degradation products 1, 2, 3, and 4. Whereas in oxidative conditions, degradation product 5 was identified. All the five degradation products formed are novel, which was not reported earlier. This is the first time documented complete structural characterization of all five degradation products by using a hyphenated analytical technique. High-resolution mass, and nuclear magnetic resonance spectroscopy were used in the present study to get concrete confirmation of degradation products structures. The current method is also used to identify degradation products with shorter runtime in the future.

## Introduction

In type 2 diabetes medication, Ertugliflozin is used as an inhibitor^1–3^. It’s available as a solitary medicine and combined with sitagliptin and metformin HCl. The available literature in the market reveals some analytical and bioanalytical technique development validation, and stability suggesting investigations are accessible with the drug (Ertugliflozin) and the mixture of sitagliptin and metformin. The medication Ertugliflozin (brand name Steglatro) is used to treat type 2 diabetes. The Food and Drug Administration authorized it for use as a monotherapy and in a fixed dosage combination with either sitagliptin or metformin in the United States^4^. It was approved for use as a monotherapy or combination treatment in Europe in March 2018. Ertugliflozin belongs to the family of medicines known as gliflozins and is SGLT2 inhibitor. Segluromet is sold in conjunction with metformin, and Steglujan is offered in combination with sitagliptin.

**Figure 1:**
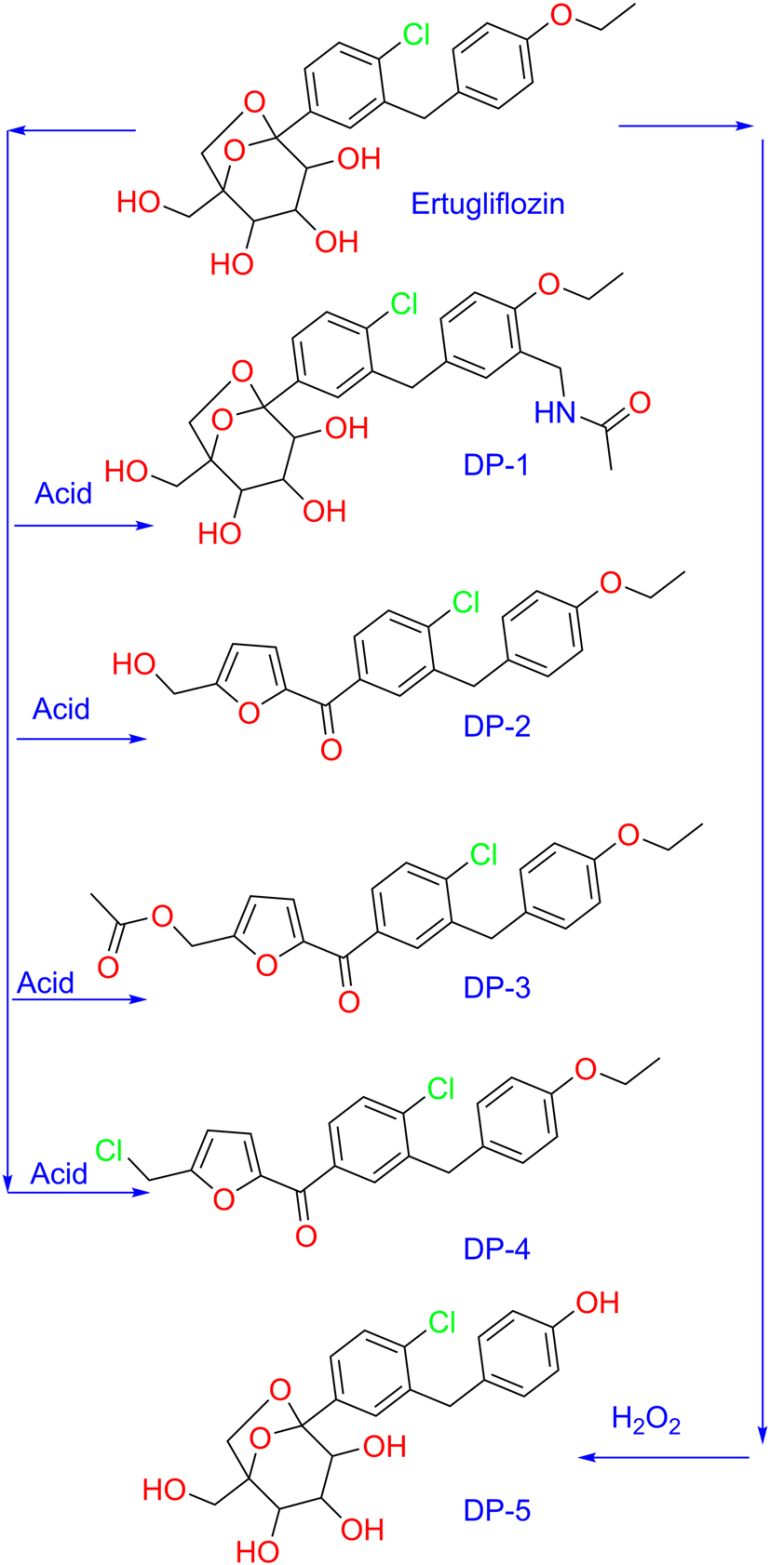
Structures of Ertugliflozin and its degradation products.

Ertugliflozin is marketed under the brand name Steglatro. Its molecular formula is C_22_H_25_ClO_7_ (Mol. Wt.: 436.13) and the chemical name is 5-(4-chloro-3-(4-ethoxybenzyl)phenyl)-1-(hydroxymethyl)-6,8-dioxabicyclo[3.2.1]octane-2,3,4-triol. Ertugliflozin is in pyroglutamic acid salt form and appears as a white non-hygroscopic crystalline powder. It is soluble in acetone and ethanol, slightly soluble in acetonitrile and ethyl acetate, and only in water sparingly soluble. Figure 1 summarizes the structure of Ertugliflozin and its degradation products. Scrutinizing the literature discovered few publications on the analysis of Ertugliflozin unique and combined with metformin and sitagliptin. Ertugliflozin strategy development was reported for its combination with metformin RP-HPLC^5–11^, with sitagliptin^12–18^, in combination with both metformin and sitagliptin^19^. Several bioanalytical strategies utilizing LC–MS/MS approaches have been reported to detect sitagliptin in plasma and Ertugliflozin^20,21^. However, until now, there was no literature available to explain the Ertugliflozin degradation products and their characterization. None of the literature provided the NMR, high-resolution mass spectrometry, and IR studies of the degradation products. The present study explains the detailed structural characterization for all 5 degradation products of Ertugliflozin. Hence, the current research was endeavored to provide concrete evidence for the degradation products structures, which involved the chromatographic method development of UHPLC-MS method for ERG and its 5 degradation products, well resolved in 4 min runtime along with HRMS/MS, IR, and NMR (1D, 2D) experiments.

## Material and methods

### Chemicals and reagents

Ertugliflozin active pharmaceutical gift sample was obtained from leading pharma organizations in Hyderabad. Reagents and chemicals used for the research were sodium hydroxide, hydrochloric acid, 30% hydrogen peroxide (analytical grades) formic acid (LCMS grade), acetonitrile (HPLC grade), procured from Honeywell Research Chemicals, India. The water used for the analysis was from a milli-Q instrument from Millipore, Amsterdam, Netherlands. Dimethylsulfoxide-d6 (NMR grade) from Cambridge Isotope Laboratories, Inc. D, 99.9% + 0.03% v/v.

### Instrumentation and software

The analytical instruments and supported software engaged for the present work are UHPLC-MS instrument from Acquity UHPLC frontend with waters single quadrupole detector with and maslynx 4.2 software. HRMS instrument from Thermo with Dionex ultimate 3000 LC frontend q-exactive orbitrap MS with ESI ion source and x-calibur software. Purification instrument from Waters binary module 2545, detector-2489 and autosampler 2707 with chrom scope-2.1 software. NMR instrument from Bruker Avance neo 400 MHz with topspin 4.09 software. Analytical balance from Sartorius-SQP-F, and Infrared spectroscopy instrument from Shimadzu ir-afinity-1S and lab solution software.

### Ultra-high performance liquid chromatography-mass spectrometry (UHPLC-MS) method development (optimization of chromatographic conditions)

Liquid chromatography resolution was achieved on Waters make Single Quadrupole mass Detector (SQD2) attached with Acquity UHPLC & Photo diode array detector (PDA) front-end. Dual polarity- negative and positive with ESI (electrospray ionization) source with waters single quadrupole mass spectrometer was utilized for mass analysis MS optimization was done by using scan mode from 100 to 1200 Daltons (Da). The desolvation temperature was kept at 350 °C. 700 L h^−1^ gas was set for the flow of desolvation and that of cone gas was set at 60 L h^−1^. The liquid chromatography-mass spectrometer instrument was operated using MassLynx 4.1 application manager. Samples were maintained at 10 °C temperature and were run with shorter chromatographic runtime of 4.0 min and an injection volume of 0.5 µL.

**Figure 2:**
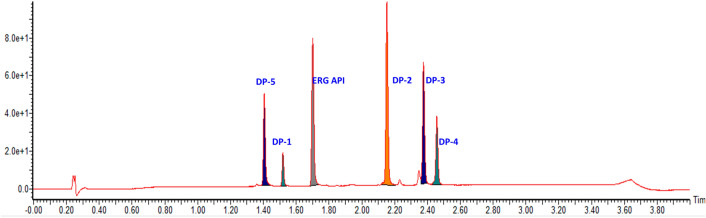
Method development of Ertugliflozin and its degradation products in acquity BEH C18 column.

**Figure 3:**
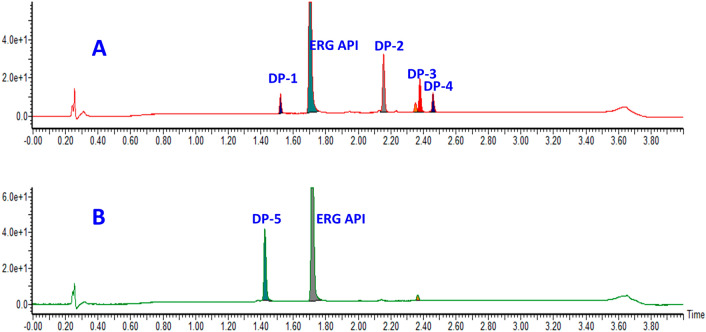
Ertugliflozin degradation pathway acid hydrolysis (**A**) and oxidative hydrolysis (**B**).

In pursuit of an appropriate, reliable, and reproducible method, different buffers using different columns were screened to optimize the resolution. Initially, the method development was done using trifluoroacetic acid, formic acid, ammonium bicarbonate buffers, and different columns like acuity phenyl, C18, and CSH C18. During copious trials, 0.1% formic acid buffer with acquity BEH C18 showed positive and encouraging results and the remaining trials got either poor resolution or resolution. Further, extensive trials were made on acquity BEH column with various flow rate and gradient conditions. The optimum separation of all degradation products and API was achieved on the Waters Acquity UPLC BEH C18 column (50 × 2.1 mm, 1.7 µm) column. The mobile phase included mobile phase-A and B having 0.1% formic acid in water and 0.1% formic acid in acetonitrile. Separation was attained using gradient program Time (min)/B conc (%): 0/3, 0.5/3, 2.5/98, 3.5/98, 3.6/03, 4/03 at a flow rate of 0.4 mL min^−1^ and column temperature of 35 °C. A photo diode array (PDA) detector was used to monitor the eluents. The diluent used in the ratio of 1:1 (v/v) mixture of water and acetonitrile. All 6 peaks were separated with good peak shape and resolution in this condition. The final method development trace is shown in Fig. 2, and degradation traces are shown in Fig. 3.

### High-resolution mass spectrometry (HRMS)

**Figure 4:**
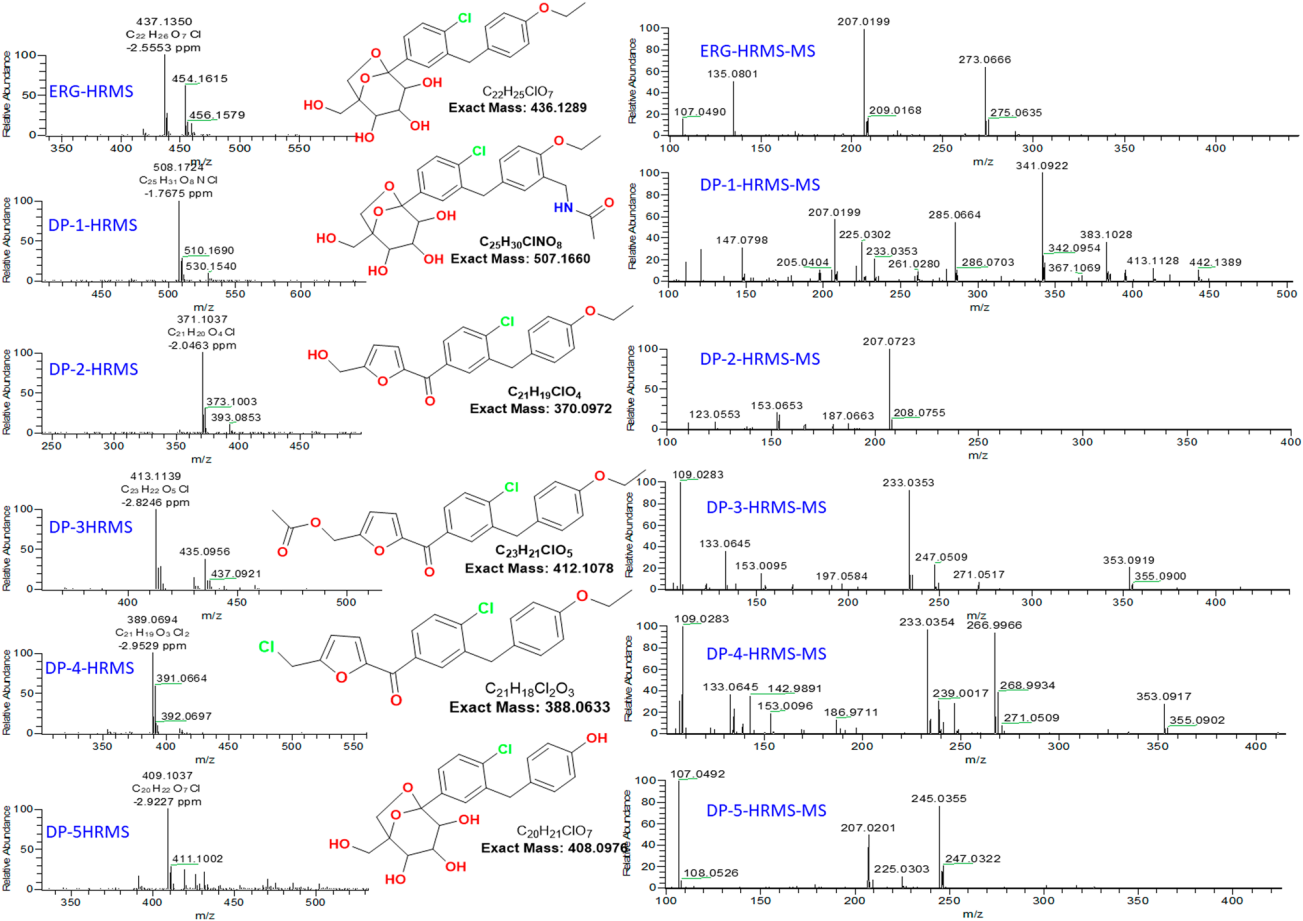
HRMS & HRMS-MS data for Ertugliflozin and its degradation products.

Samples were analysed using an ESI source equipped with Thermo Q Exactive orbitrap MS; UHPLC Dionex Ultimate 3000 with PDA detector as front-end. Utilized instrument source parameters were Spray Voltage: 3.5 kV; Aux gas flow rate: 14; Capillary Temperature: 270 °C; Sweep gas flow rate: 3; Aux gas heater Temperature: 440 °C. Sheath gas flow rate: 53. Mass data were acquired using xcalibur software. Reserpine (monoisotopic mass: 608.2734 Da) was used as a standard to check the mass analyser accuracy. Chromatographic conditions were like UHPLC-MS. The HRMS data for all degradation products are shown in Fig. 4. The mass fragmentation data obtained in HRMS-MS analysis for all degradation products are displayed in Table 1.

**Table 1:** HRMS and MS/MS data for Ertugliflozin and its degradation products.

Products name	Formula	m/z calculated	m/z obtained	Mass error (ppm)	Fragments (m/z)
Ertugliflozin	C_22_H_25_ClO_7_	437.1367	437.1350	− 2.5835	273, 207, 135, 107
DP-1	C_25_H_30_ClNO_8_	508.1738	508.1724	− 1.7675	383, 341, 285, 225, 207, 147, 121, 111
DP-2	C_21_H_19_ClO_4_	371.1050	371.1037	− 2.0463	207, 153
DP-3	C_23_H_21_ClO_5_	413.1156	413.1139	− 2.8246	353, 247, 233, 153, 133, 109
DP-4	C_21_H_18_Cl_2_O_3_	389.0711	389.0694	− 2.9529	353, 267, 233, 153, 133, 109
DP-5	C_20_H_21_ClO_7_	409.1054	409.1037	− 2.9227	245, 207, 107

### Preparative HPLC and sample preparation for purification

Waters semi-preparative HPLC 2489 dual UV detector, 2545 pump module, and 2707 sample manager with auto fraction collector-III. The total instrument was controlled with ChromScope-2.1 software, and in-house packed Luna C18 150 × 25 mm, 5 µm was used to isolate the degradation products. All isolated pure fractions were lyophilized using Lyofreeze lyophilizer.

Degradation was seen in acidic and peroxide environments. To neutralize the acid hydrolyzed sample, a saturated solution of (NH_4_)_2_CO_3_ was utilized, and the resulting solution was solidified using lyophilization. After diluting the peroxide solution, evaporated to get a free solid. For preparative HPLC purification, the identical sample was dissolved in a small amount of the mobile phase.

### Nuclear magnetic resonance spectroscopy (NMR)

^1^H, ^13^C, and 2D NMR spectra of Ertugliflozin and degradation products were analyzed in DMSO-d6 solvent on Bruker Avance Neo 400 MHz NMR instrument furnished with 5 mm broad band observe probe (BBO) with shim system of Z-gradient with sensitivities of 225:1 and 480:1. TMS (tetramethyl silane) signal at zero ppm was used as a reference for ^1^H and 39.5 ppm septet signal of DMSO-d6 for ^13^C.

### FT-IR spectroscopy

Shimadzu IR-Afinity-1S model with lab solutions software was used to recognize the functional groups like alcohol, ketone and chloro ketone present in the compounds. KBr was used as a dispersion medium to prepare the sample pellets.

### Degradation trend of Ertugliflozin

As per ICH stability guidelines^22–26^, various Forced degradation parameters were employed, i.e., thermal, photolytic oxidation, neutral, acid, and base hydrolytic conditions. Forced degradation study of ERG was performed as mentioned in the ICH guidelines for stability study. The ERG standard solution (10 mg mL^−1^), One milliliter of the solution was transferred into a 10 mL volumetric flask and diluted up to the mark with 30% hydrogen peroxide solution. The solution was stirred at room temperature. One milliliter of the solution was taken and make up to 10 mL with ACN water (1:1 v/v). To obtain a final concentration of 100 µg mL^−1^, it was taken for further studies to get the degradation behavior. For photodegradation, 25 mg of ERG sample was exposed to 254 nm UV light for 48 h in UV chamber. For thermal degradation, 25 mg of ERG sample was kept at 100 °C for 48 h and it was used for further studies to observe the degradation behaviour.

For neutral, acid and base hydrolysis study, a standard stock solution of ERG (10 mg mL^−1^) was prepared in ACN and water (1:1 v/v). 1 mL of ERG standard stock solution was transferred to a 10 mL volumetric flask. Then make up to the mark using water for neutral degradation, 1 N NaOH for alkaline degradation and 1 N HCl for acidic degradation (Initially, the same procedure was performed with 0.1 N NaOH and 0.1 N HCl. However, considerable degradation was not observed, so the strength of acid and base were increased to 1 N). The solution was kept stirring at 60 °C temperature. One milliliter of the solution was taken, neutralized, and transferred to 10 mL volumetric flask. Then the volume was makeup to mark with water to obtain a final concentration of 100 µg mL^−1^. It was taken for further studies to get the degradation behavior.

ERG compound is highly stable in neutral, basic hydrolysis, photolytic and thermal, conditions and did not show any degradation, which confirms the stability of ERG under the above conditions. The drug was found to be labile to acid hydrolysis and peroxide conditions. As a result, ~ 15–20% of degradation was observed in both peroxide (30% H_2_O_2_ with stirring at room temperature, up to 48 h) acid (1 N HCl with reflux at 60 °C stirring temperature, up to 48 h) and conditions. Detailed degradation conditions and results are displayed in Table 2, and degradation chromatograms of acid hydrolysis and peroxide conditions are shown in Fig. 3. In acid hydrolysis conditions, 4 DPs were formed (DP-1, DP-2, DP-3 and DP-4), whereas, in oxidative condition, one degradative product (DP-5) was formed. The confirmed structures of all the DPs are revealed in Fig. 1.

**Table 2:** Ertugliflozin forced degradation studies.

Conditions	% area of degradation product					
	DP-1	DP-2	DP-3	DP-4	DP-5	API
Ertugliflozin API	–	–	–	–	–	99
Acid (1N HCl stirring at 60 °C up to 48 h)	3.7	13.4	7.8	4.5	–	68
Base (1N NaOH stirring at 60 °C up to 48 h)	–	–	–	–	–	98
Neutral (Water stirring at 60 °C up to 48 h)	–	–	–	–	–	98
Oxidation (30% H2O2 stirring at rt up to 48 h)	–	–	–	–	24.4	73.5
Thermal (explore to 100 °C up to 48 h)	–	–	–	–	–	98
Photolytic (explore at 254 nm for 48 h)	–	–	–	–	–	98

The formation of degradation impurities was as follows, for the Formation of DP1, Initial acid mediated electrophilic substitution of protonated formaldehyde to the ortho position as in compound 1 followed by Ritter chemistry with acetonitrile afforded DP1. As per the literature of Journal of Analytical and Applied Pyrolysis, 113 (2015) 621–629^27^, sugar moiety in the target molecule undergoes gradual decomposition to form formaldehyde under hot acidic condition. This upon react with the target molecule under acidic medium (as given in the manuscript) promotes the formation of 3. This in turn react with acetonitrile (used as solvent for the solubility purpose) to form DP-1 through Ritter chemistry.

Formation of DP2, the proposed degradation pathway for the formation of DP2 is based on the literature (Chemical Engineering Journal 307, 2017, 877–883)^28^ under Bronsted acidic condition.

Formation of DP3/DP4, as per the literature of Journal of Analytical and Applied Pyrolysis, 113 (2015) 621–629, sugar moiety in the target molecule undergoes gradual decomposition to form formaldehyde/acetaldehyde and acetic acid in different proportions^29,30^. The formed acetic acid involved in the esterification of the DP2 under the existing hot acidic condition. In a similar way, the free hydroxymethyl group undergoes chlorination via SN2 mechanism. Accordingly, the acid protonates the hydroxyl group and is successively displaced by the chloride to form the corresponding DP4.

Formation of DP5, Th proposed mechanism for the formation of DP5 is through the oxidative cleavage of aryl ethoxy group to form the corresponding phenol i.e., DP5.

## Results and discussion

Using mass spectrometry (UHPLC-MS), individual samples were analysed to determine the outcomes of all the stress studies. All degradants formed over a time, and from each constraint, study conditions are outlined in the experimental section. Five substantial degradation products were identified, isolated, and characterized by UHPLC-MS, Prep-HPLC, HRMS, 2D-NMR and FTIR techniques. Most of these degradation products are formed by the rearrangement of six-member carbohydrate ring of API into five-member furan ring.

### Isolation of degradation products

The degradation products formed as considerable percentage of > 5% as a result of degradation was observed under peroxide and acid stress conditions. Luna C18 column (150 mm × 25 mm, 5 μm) and acetonitrile, 0.1% formic acid in aqueous were used as a mobile phase for purification. Consecutive injections of crude sample solutions were injected, and the fractions collection was done based on UV response and later mass confirmed by LC–MS. Collected separately fractions of various degradation products and lyophilized to get a free solid.

### Structural confirmation of degradation products

**Figure 5:**
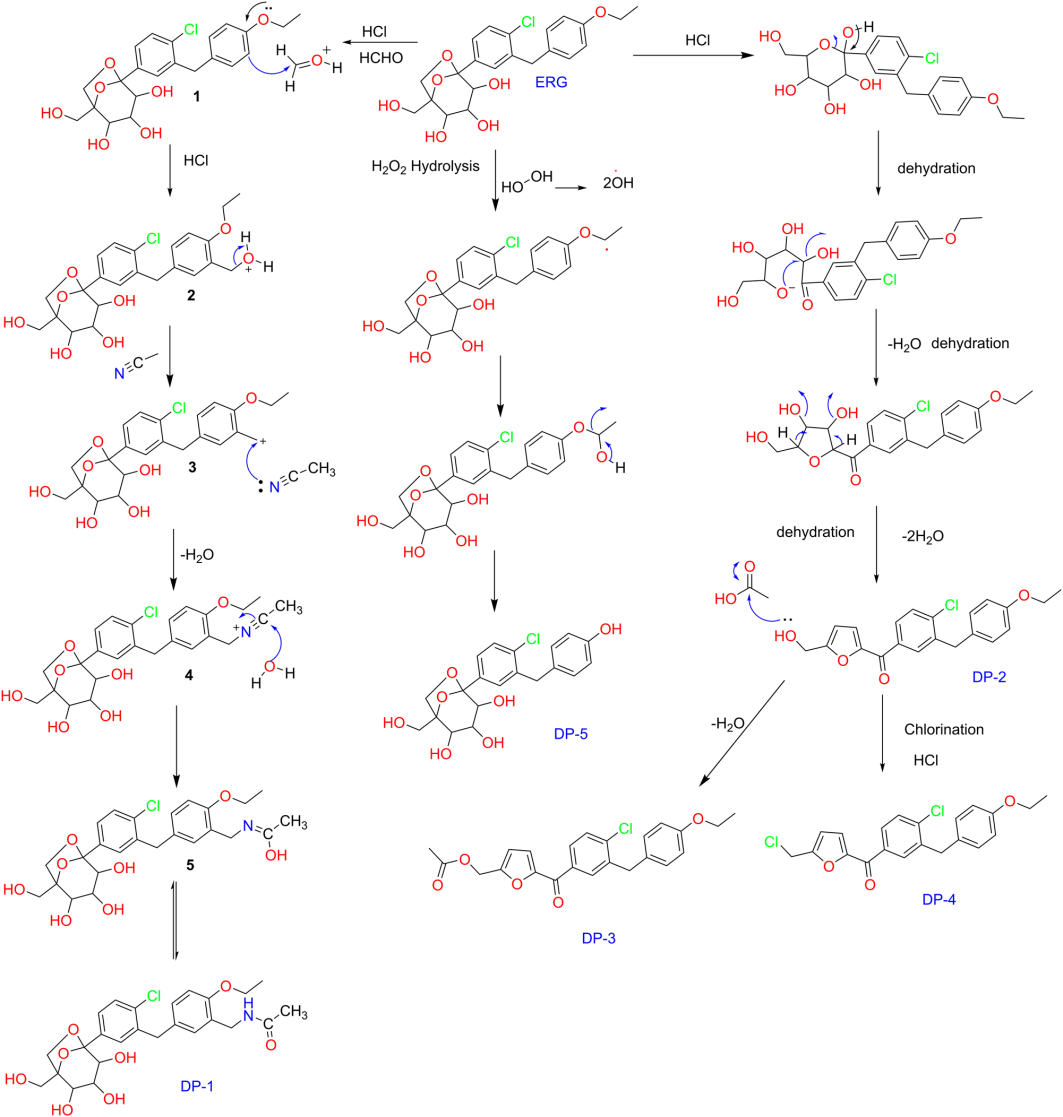
Proposed degradation behavior of Ertugliflozin in acidic hydrolytic and peroxide conditions.

**Figure 6:**
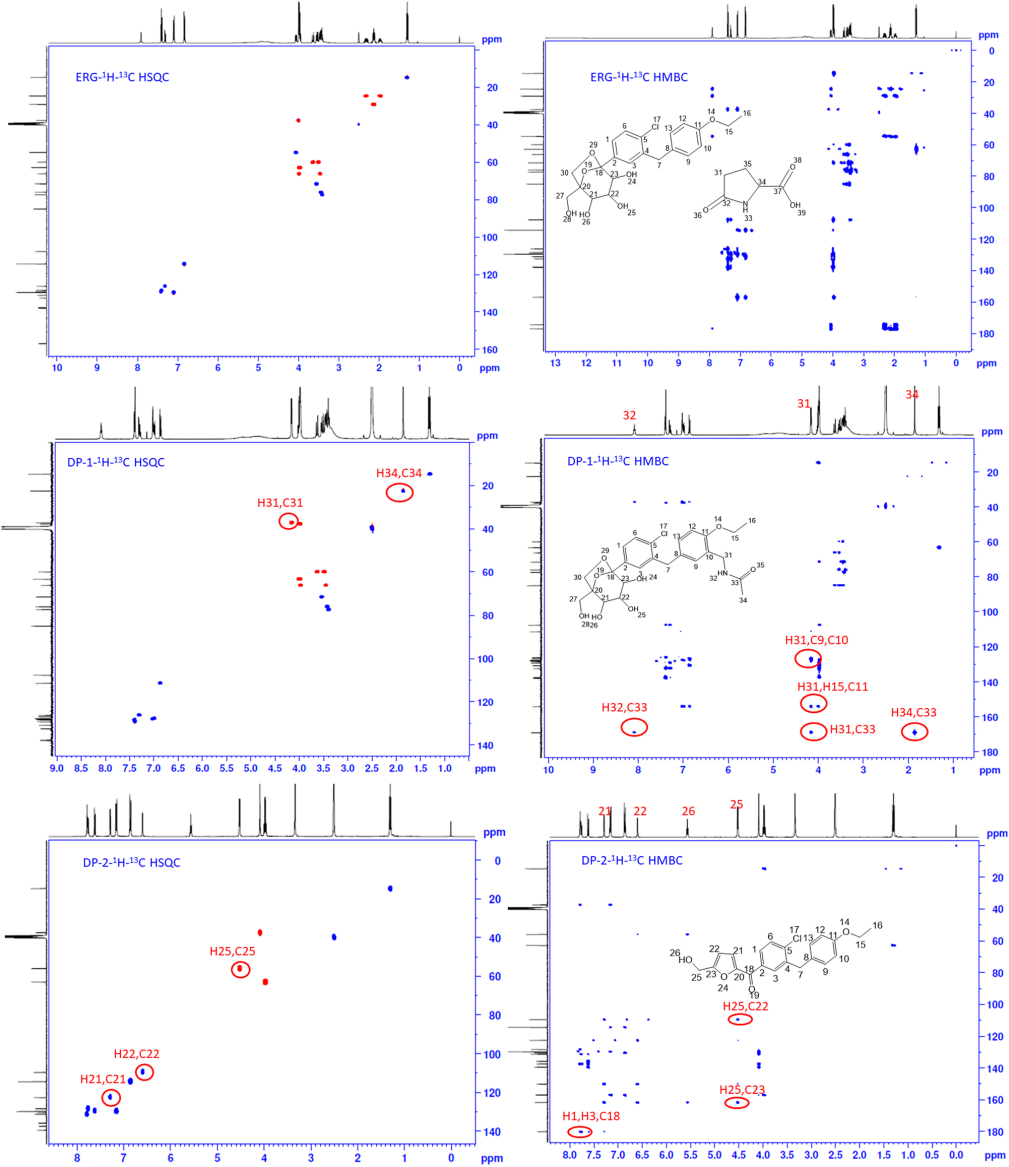
Illustrative NMR spectrums for ERG, DP-1 and DP-2.

To get the structural information of Ertugliflozin API, all analytical data was recorded for reference purposes. Under ESI–MS positive mode conditions, [M + H]^+^ and ammonia adduct [M + H + NH_3_]^+^ ions were detected as 437.1350 and 454.1617, respectively, which confirms the molecular formula for Ertugliflozin is C_22_H_25_ClO_7_. The confirmed data of Ertugliflozin is mentioned at HRMS, HRMS/MS spectrum (Fig. 4, Table 1), IR (Table 4). HRMS/MS proposed tentative mechanism for protonated fragmentation ions of ERG are shown in supplementary data Fig. S1. The proposed structural mechanism to explain the degradation behaviour of Ertugliflozin in acidic hydrolytic and peroxide conditions is shown in Fig. 5, and the structural conformation of ERG using 2D-NMR is shown in Figs. 6 and S2. The structural conformation of ERG is done by HRMS and 2D-NMR. All the other degradant products are characterized based on the comparison of this structural elucidation data.

### Degradation product (DP-1) characterization

The Ertugliflozin treatment with acid resulted in degradation product-1 (DP-1). This degradation product was isolated, and its mass was confirmed as [M + H]^+^ 508.1724 (− 1.7675 ppm error) in HRMS for the molecular formula C_25_H_30_ClN_5_O_8_ shown in Fig. 4. The HRMS spectrum confirms the presence of one chlorine atom in the structure. The complete structure of the degradation product was determined by NMR and HRMS studies. The sample was prepared in DMSO-d6 solvent and subjected to NMR analysis. Initially recorded ^1^H NMR and D_2_O exchange experiments. On comparison of ^1^H and D_2_O data confirm the presence of five exchangeable protons in the compound and in aromatic region missing of 1,4 disubstituted ring pattern and presence the pattern of 1,2,4 trisubstituted ring. The key difference from API compound is the presence of secondary amide (H32), methylene protons (H31) and acetyl group (H34). In ^1^H NMR, a total of 30 protons were present, the secondary amide is at 8.08 ppm, and aromatic protons were shown between 6.0 and 8.0 ppm. Primary and secondary alcoholic protons are at 4.5–5.5 ppm, and aliphatic protons resonance between 1.0 and 4.5 ppm. *N*-methylene protons are showing at 4.15 ppm and acetyl protons are at 1.85 ppm. *O*-methylene is at 4.00 ppm, and aromatic methylene protons are at 3.97 ppm. In ^13^C NMR a total of 25 carbons were showing; most downfield carbon is acetamide carbonyl showing at 169.26 ppm, followed by *O*-ethyl group attached quaternary carbon is at 154.36 ppm. The remaining aromatic carbons were between 100 and 140 ppm, aliphatic carbons were showing at 10–90 ppm. In HSQC experiment confirmed as H31 methylene protons attached carbons is showing at 37.16 ppm, and *N*-acetyl protons attached carbon is showing at 22.47 ppm. In the gDQ-COSY experiment, H31 protons showed correlation with the H32 proton. In ^1^H-^15^N HSQC experiment H32 proton was confirmed as an amidic NH proton, and its nitrogen value is showing at 115 ppm. In ^1^H-^13^C HMBC experiment, H31, H32, and H34 protons showed connectivity to C33 carbon, and H31 protons showed connectivity to C9, C10 and C11 carbons. All the key information of 1D and 2D NMR data confirms that 1,4 disubstituted ring converted to benzylacetamide. Finally, it formed *N*-(5-(2-chloro-5-(2,3,4-trihydroxy-1-(hydroxymethyl)-6,8-dioxabicyclo[3.2.1]octan-5-yl)benzyl)-2-ethoxybenzyl)acetamide and the 2D NMR spectrum was depicted in Figs. 6 and S3. The chemical shifts were in Table 3 and the proposed degradation mechanism was shown in Fig. 5.

**Table 3:** Relative NMR assignments for Ertugliflozin and its degradation products.

ERG-API				DP1				DP-5			
**Atom no**	Type of atom	^1^H chemical shift (ppm) coupling const (J)	^13^C Chemical Shift (ppm)	Atom no	Type of atom	^1^H chemical shift (ppm) coupling const (J)	^13^C chemical shift (ppm)	Atom no	Type of atom	^1^H chemical shift (ppm) coupling const (J)	^13^C chemical shift (ppm)
1	CH	7.29(dd,8.4Hz,2.0Hz,1H)	126.24	1	CH	7.28(dd,8.4Hz,2.0Hz,1H)	126.19	1	CH	7.27(dd,8.4Hz,2.4Hz,1H)	126.14
2	C	–	138.08	2	C	–	138.05	2	C	–	138.02
3	CH	7.40(d,2.0Hz,1H)	129.28	3	CH	7.38(d,2.4Hz,1H)	129.23	3	CH	7.37(s,1H)	129.22
4	C	–	137.70	4	C	–	137.60	4	C	–	137.93
5	C	–	132.59	5	C	–	132.51	5	C	–	132.56
6	CH	7.38(d,8.4Hz,1H)	128.44	6	CH	7.39(d,8.4Hz,1H)	128.38	6	CH	7.39(d,8.4Hz,1H)	128.41
7	CH_2_	3.99(s,2H)	37.66	7	CH_2_	3.97(s,2H)	37.71	7	CH_2_	3.93(s,2H)	37.7
8	C	–	131.14	8	C	–	130.82	8	C	–	129.45
9,13	CH	7.08(d,8.8Hz,2H)	129.58	9	CH	7.02(s,1H)	127.96	9,13	CH	6.97(d,8.4Hz,2H)	129.57
10,12	CH	6.81(d,8.8Hz,2H)	114.31	10	C	–	126.93	10,12	CH	6.61(d,8.4Hz,2H	115.2
11	C	–	156.92	11	C	–	154.36	11	C	–	155.65
15	CH_2_	3.97(q,7.2Hz,2H)	62.88	12	CH	6.85(d,8.4Hz,1H)	111.41	14	OH	9.24(s,1H)	–
16	CH_3_	1.29(t,7.2Hz,3H)	14.68	13	CH	6.97(dd,8.4Hz,1.6Hz,1H)	127.73	16	C	–	107.7
18	C	–	107.69	15	CH_2_	4.00(q,7.2Hz,2H)	63.27	18	C	–	85.01
20	C		85.01	16	CH_3_	1.31(t,6.8Hz,3H	14.69	19	CH	3,53(dd,7.6Hz,4Hz,1H)	71.53
21	CH	3.53(d,7.6hZ,1H)	71.54	18	C	–	107.65	20	CH	3.43(m,1H)	76.06
22	CH	3.43(t,7.6Hz,1H)	76.06	20	C		84.97	21	CH	3.41(m,1H)	77.38
23	CH	3.39(d,7.6Hz,1H)	77.38	21	CH	3.52(d,7.2Hz,1H)	71.49	22	OH	4.89(d,6.4Hz,1H)	–
24,25, 26,28	OH	4.50–5.50 (broad hump,4H)	–	22	CH	3.41(t,7.6Hz,1H)	76.02	23	OH	4.98(d,4.4Hz,1H	
27	CH_2_	3.48,3.62(d,12.4Hz,2H)	59.94	23	CH	3.38(d,7.6Hz,1H)	77.35	24	OH	5.21(d,4.8Hz,1H	-
30	CH_2_	3.47,3.97(d,6.8Hz,2H)	66.2	24,25, 26,28	OH	4.5–5.4(broad hump,4H)	-	25	CH_2_	3.49,3.62 (dd,12,4Hz,5.6Hz,2H	59.93
31	CH_2_	2.12(m,2H)	29.06	27	CH_2_	3.47,3.61(d,12.8HZ,2H)	59.89	27	CH_2_	3.46,3.96(d,7.2Hz,2H)	66.2
32	C	–	176.99	30	CH_2_	3,46,3.97(d,6.8Hz,2H)	66.15	28	OH	4.77(t,6.0Hz,1H)	–
33	NH	7.90(s,1H)	–	31	CH_2_	4.15(d,6.0Hz,2H	37.16				
34	CH	4.04(dd,8.8Hz,4.4Hz,1H)	54.75	32	NH	8.08(t,6.0Hz,1H)	–				
35	CH_2_	1.94,2.31(m.2H)	24.61	33	CO	–	169.26				
				34	CH_3_	1.85(s,3H)	22.47				

DP-2				DP-3				DP-4			
Atom no	Type of atom	^1^H chemical shift (ppm) coupling const (J)	^13^C chemical shift (ppm)	Atom no	Type of atom	^1^H chemical shift (ppm) coupling const (J)	^13^C chemical shift (ppm)	Atom no	Type of atom	^1^H chemical shift (ppm) coupling const (J)	^13^C chemical shift (ppm)
1	CH	7.75(dd,8.0hZ,2.0Hz,1H)	128.38	1	CH	7.75(dd,8.4Hz,2.0Hz,1H)	128.45	1	CH	7.76(dd,8.4Hz,1H)	128.45
2	C	–	135.81	2	C	–	135.49	2	C	–	135.45
3	CH	7.78(dd,2.0Hz,1H)	131.28	3	CH	7.81(d,2.0Hz,1H)	131.42	3	CH	7.81(d,2.0Hz,1H)	131.41
4	C	–	139.55	4	C	–	139.59	4	C	–	139.62
5	C	–	137.44	5	C	–	137.68	5	C	=	137.74
6	CH	7.61(d,8.4Hz,1H)	129.66	6	CH	7.62(d,8.4Hz,1H)	129.73	6	CH	7.62(d,8.4Hz,1H)	129.74
7	CH_2_	4.08(s,2H)	37.35	7	CH_2_	4.09(s,2H)	37.34	7	CH_2_	4.09(s,2H)	37.37
8	C	–	130.53	8	C	–	130.52	8	C	–	130.5
9,13	CH	7.14(d,8.8Hz,2H)	129.82	9,13	CH	7.14(d,8.4Hz,2H)	129.79	9,13	CH	7,15(d,8.4Hz,2H)	129.81
10,12	CH	6.84(d,8.4Hz,2H)	114.42	10,12	CH	6.84(d,8.8Hz,2H)	111.4	10,12	CH	6.86(d,8.8Hz,2H)	114.41
11	C	–	157.05	11	C	–	157.04	11	C	–	157.05
15	CH_2_	3.96(d,6.8Hz,2H)	62.87	15	CH_2_	3.94(q,6.8Hz,2H)	62.86	15	CH_2_	3.96(q,7,2Hz,2H)	62.86
16	CH_3_	1.29(t,6.8Hz,3H)	14.64	16	CH_3_	1.29(t,6.8Hz,3H)	14.62	16	CH_3_	1.29(t,7.2Hz,3H)	14.63
18	CO	–	180.11	18	CO	–	180.22	18	CO	–	180.21
20	C	–	150.15	20	C	–	150.92	20	C	–	150.07
21	CH	7.28(d,3.2Hz,1H)	122.63	21	CH	7.32(d,3.6Hz,1H)	122.12	21	CH	7.31(d,3.6Hz,1H)	122.26
22	CH	6.59(d,3.6Hz,1H)	109.6	22	CH	6.79(d,3.6Hz,1H)	112.85	22	CH	6.82(d.3.6Hz.1H)	112.62
23	C	–	161.7	23	C	–	154.92	23	C	–	155.56
25	CH_2_	4.51(d,5.6Hz,2H)	55.94	25	CH_2_	5.15(s,2H)	57.47	25	CH_2_	4.93(s,2H)	36.88
26	OH	5.56(t,6.0Hz,1H)	–	27	CO	–	169.86				
				28	CH_3_	2.07(s,3H)	20.46				

Further, stretching frequency at 3415 cm^−1^ in FT-IR spectra specifies the presence of NH and OH groups, and 1645 cm^−1^ stretching frequency directs the presence of amide keto groups. The stretching frequency at 811 cm^−1^ leads to the existence of the C–Cl group. A significant FT-IR spectrum established the existence of keto, amide, alcohol, and chloride functional groups, as presented in Table 4. For DP-1, HRMS/MS spectrum and proposed tentative mechanism for protonated fragmentation ions were shown in Figs. 4 and S1.

**Table 4:** Comparative FTIR data of Ertugliflozin and its degradation products.

Assignment	Region (cm^−1^)	API	DP-1	DP-2	DP-3	DP-4	DP-5
O–H/N–H stretching	3700–3300	~ 3534	~ 3415	~ 3481,3334	–	–	~ 3443
C=O stretching	1760–1630	~ 1735, 1660 (salt)	~ 1645	~ 1637	~ 1744, 1645	~ 1649	–
C–H bending	1600–1450	~ 1513	~ 1504	~ 1511	~ 1511	~ 1511	–
C–O/C–N stretching	1370–1000	~ 1369, 1329	~ 1248, 1085, 1038	~ 1244,1043	~ 1349, 1039	~ 1305, 1246	~ 1268
C–Cl	850–550	~ 826	~ 811	~ 756	~ 754	~ 841, 703	~ 755

### Degradation product (DP-2) characterisation

The DP-2 compound was formed in acid hydrolysis of the ERG compound. To acquire structural information, DP-2 HRMS analysis was performed and 371.1037 [M + H]^+^ was achieved as a protonated molecule with − 2.0463 ppm error for the calculated molecular formula C_21_H_19_ClO_4_ shown in Fig. 4. The HRMS spectrum confirms the presence of one chlorine atom in the structure, and the mass of the degradation product has 66 units less than parent Ertugliflozin. To obtain the meticulous structure of the degradation product 1D and 2D NMR experiments have been performed. The ^1^H NMR data of DP-2 showed extreme differences from API compound. Here we could not see the bicyclic ring protons and noticed the primary alcohol proton at 5.56 ppm as a triplet, it was exchanged in D_2_O exchange. The methylene protons show as a doublet at 4.51 ppm, furan ring protons showing at 6.59 and 7.28 ppm with J value 3.2Hz. In aromatic region 1,4 disubstituted ring, 1,2,4 trisubstituted pattern protons were present like parent compound at 6–8 ppm, and in aliphatic region *O*-ethoxy protons, methylene protons were shown between 1.0 and 4.5 ppm region. In ^13^C NMR, a total of 19 carbons were shown due to symmetry C9–C13 and C10–C12 carbons showing each as one signal. Most downfield carbon is a ketone, and it is resonating at 180.11 ppm. Quaternary carbons of furan ring are showing at 150.15, 161.7 ppm, and *O*-ethoxy attached aryl carbon is at 157.05 ppm; remaining aryl carbons are showing at 110–140 ppm. In the aliphatic region 4 carbons were showing, key methylene carbon is at 55.94 ppm, by HSQC experiment confirmed the same. Other key information from HSQC experiment is identifying the furan ring protonated carbons. From COSY experiment, primary alcohol proton H26 showed a correlation with H25 methylene protons, and furan ring protons H21, H22 are shown correlation with each other. From g-HMBC experiment confirms H25 protons showing connectivity to C22 and C23 carbons and H1, H3 protons show connectivity to C18 carbon. Based on NMR data concluded that bicyclic ring has been rearranged and formed 2,5 substituted ring furan rings as structure depicted in Figs. 6 and S4 and formed (4-chloro-3-(4-ethoxybenzyl)phenyl)(5-(hydroxymethyl)furan-2-yl)methanone. The chemical shifts were in Table 3 and proposed degradation mechanism was shown in Fig. 5.

Further, stretching frequency at 3481 cm^−1^ in FTIR spectra confirms the presence of OH groups, and 1637 cm^−1^ stretching frequency specifies the presence of keto groups. The stretching frequency at 756 cm^−1^ directs the presence of the C–Cl group. FT-IR significant frequencies established the existence of alcohol, keto, and chloro functional groups, as depicted in Table 4. For DP-2, HRMS/MS spectrum and proposed tentative mechanism for protonated fragmentation ions were shown in Figs. 4 and S1.

### Degradation product (DP-3) characterization

**Figure 7:**
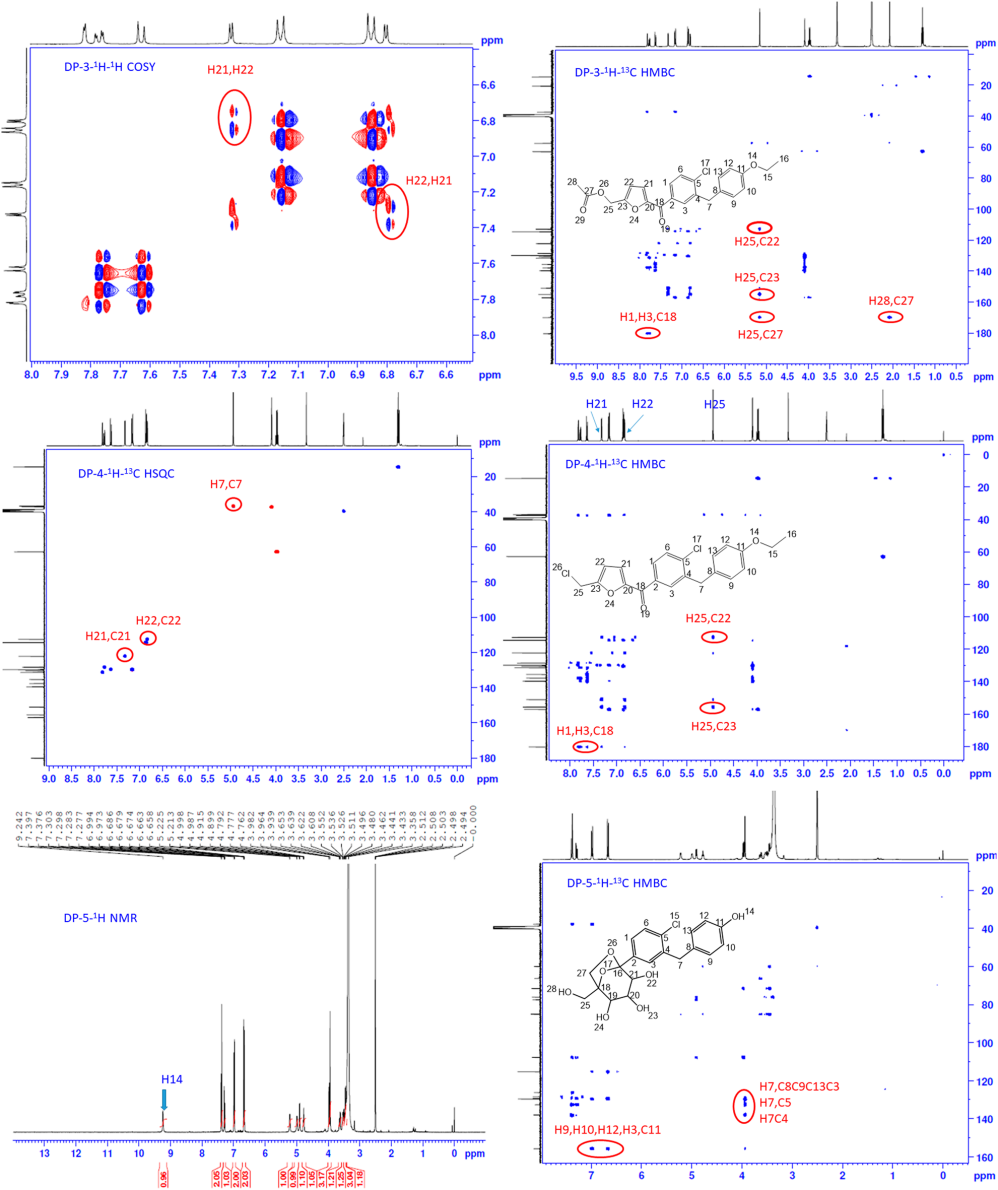
Illustrative NMR spectrums for DP-3, DP-4 and DP-5.

Acid hydrolysis of ERG compound resulted in the DP-3 compound. To acquire structural information_ of DP-3, HRMS analysis was executed and observed protonated molecule [M + H]^+^ as 413.1139 with − 2.8246 ppm error, which confirms the calculated molecular formula C_23_H_21_ClO_5_ shown in Fig. 4. The mass of the degradation product has less by 24 units than that of ERG, and to know the exact structure. Additionally, NMR experiments were recorded. DP-3 compound 1D NMR is almost like the DP-2, observed little difference, instead of alcoholic proton seen acetyl group. In ^1^H NMR, a total 21 number of protons are present, with those 9 protons from the aromatic region and furan ring protons shown at 6.79 and 7.32 ppm with J = 3.6Hz. Aliphatic region, a total 12 number of protons were present, in that 3 methylene groups are showing between 3.5 and 5.5 ppm, and acetyl group protons were showing at 2.07 ppm. In ^13^C NMR number of carbon count is 21, in those 16 carbons from aromatic and 5 carbons from aliphatic region. The most downfield carbon is ketone at 180.22 ppm, followed by *O*-acetyl is at 169.86 ppm, and *O*-ethoxy attached aryl carbon at 157.05 ppm. The furan ring quaternary carbons are at 150.92 and 154.92 ppm, protonated carbons are at 122.12 and 112.85 ppm, remaining carbons were shown between 110 and 140 ppm, aliphatic carbons were shown at 10–65 ppm. To identify protonated carbons from total number of carbons, conducted HSQC experiment and it confirms that *O*-methylene carbons C25 is 57.47 ppm and C15 is 62.86 ppm and furan ring protonated carbons were at 122.12 and 112.85 ppm. In COSY experiment H21, H22 protons show correlations. To check ^2^J and ^3^J correlations DP-3 compound HMBC experiment helped a lot, here the key correlations were H25, H28 protons are showing connectivity to C27 carbon, H25 showed connectivity to C22, C23 carbons also and H1, H3 protons showing connectivity to C18 carbon along with these remaining connectivity’s are fitting to the structure. The structure of degradation product-3 was (5-(4-chloro-3-(4-ethoxybenzyl)benzoyl)furan-2-yl)methyl acetate, and the 2D NMR spectrum was depicted in Figs. 7 and S5. The chemical shifts were in Table 3 and the proposed degradation mechanism was shown in Fig. 5.

Further confirmation done with FT-IR and 1744 and 1645 cm^−1^ stretching frequency indicates the presence of keto groups, and signals at 1349, 1039 cm^−1^ confirm the presence of C–O groups. No signal in the region of 3400–3000 cm^−1^ confirms that OH and NH protons are not present in the structure. The stretching frequency at 756 cm^−1^ directs the presence of the C–Cl group. Significant FT-IR spectrum confirmed the presence of keto, and chloro functional groups, as tabulated in Table 4. For DP-3, HRMS/MS spectrum and proposed tentative mechanism for protonated fragmentation ions are shown in Figs. 4 and S1.

### Degradation product (DP-4) characterisation

DP-4 resulted in treating ERG with acid. After isolation, HRMS was recorded to know its mass. For structural information of DP-4, HRMS analysis was performed and obtained the protonated molecule [M + H]^+^ 389.0694 with − 2.9529 ppm error for the molecular formula C_21_H_18_Cl_2_O_3_ shown in Fig. 4 and the HRMS spectrum having the dichloro pattern which conforms the compound having two chlorine atoms. It has 48 units less in comparison with the parent ERG compound. NMR studies deduced the precise structure of the DP-4. The sample was prepared in DMSO-d6 solvent and the required 1D and 2D NMR analyses were conducted to find out the structure of DP-4. In ^1^H NMR data shows 18 protons, and a minor difference was observed between ^1^H NMR data of DP-2 and DP-4 i.e., missing of the alcoholic proton, in DP-4, H25 methylene is showing as a singlet at 4.93 ppm. The furan ring protons were shown at 6.82 and 7.31 ppm with J = 3.6Hz. Two strong doublets H9, H13, and H10, H12 are showing at 7.15, 6.86 ppm, H1, H2, and H3 protons are between 7.5 and 7.9 ppm, and aliphatic protons are showing between 1 and 5 ppm. In ^13^C NMR number of carbon count is 19, due to symmetric nature in 1,4-disubstituted ring instead of 6 observed 4 signals. Most downfield carbon is ketone is resonating at 180.21 ppm, rest of the carbons showing at 110–160 ppm. In aliphatic region four carbons were showing, the key methylene carbon at C25 is at 36.88 ppm, *O*-ethoxy carbons are at 14.63 and 62.86 ppm, methylene carbon C7 is at 37.37 ppm. By HSQC experiment validated the protonated carbons, furan ring protonated carbon shifts showing at 122.26 and 112.62 ppm. In HMBC experiments observed, two important connectivity’s in that H25 proton showing ^2^J correlation with C23 carbon and ^3^J correlation with C22 carbon. The second important connectivity is H1, H3 protons showing connectivity C18 carbon. All important information of NMR data confirms that bicyclic ring has been rearranged and 1,5 substituted furan ring has been formed. As a result, the structure forming (4-chloro-3-(4-ethoxybenzyl)phenyl)(5-(chloromethyl)furan-2-yl)methanone and the 2D NMR spectrum was depicted in Figs. 7 and S6. The chemical shifts were in Table 3 and the proposed degradation mechanism was shown in Fig. 5.

FTIR spectrum indicates the presence of signal 1649 cm^−1^ indicates the presence of the keto group, and signals at 1305 and 1246 cm^−1^ indicate the presence of C–O groups. The stretching frequency at 841,703 cm^−1^ directs the presence of the C–Cl group. No single in the region of 3400–3000 cm^−1^ confirms that OH and NH protons are not present in the structure. FT-IR signals in spectrum conforms the structure includes of Chloro, and keto functional groups, as shown in Table 4. For DP-4, the HRMS/MS spectrum and proposed tentative mechanism for protonated fragmentation ions are shown in Figs. 4 and S1.

### Degradation product (DP-5) characterization

Degradation product-5 was formed by treating the API compound with Hydrogen peroxide. For DP-5 structural information, HRMS analysis was performed and observed the protonated molecule [M + H]^+^ of 409.1037 with − 2.9086 ppm error for molecular formula C_20_H_21_ClO_7_ shown in Fig. 4. From the mass data, it is understood that 28 units were less than the parent API compound and had one chlorine atom in the structure. NMR studies have done the complete characterization of DP-5. We have recorded obligatory NMR experiments for a sample dissolved in DMSO-d6. Primary experiments of NMR ^1^H and D_2_O saying that, in ^1^H-NMR data showing 21 protons, in D_2_O exchange experiment 5 protons were exchanged and showing 16 protons. Hence confirmed the presence of five labile protons in the compound. The major difference from DP-5 compound to the parent compound is missing of O-ethoxy group and the presence of phenolic OH proton at 9.24 ppm; remaining 4 labile protons are showing between 4.5 and 5.5 ppm (1° and 2° alcoholic). In aromatic region 1,4 disubstituted ring protons are at 6.66 and 6.97 ppm, chloro substituted ring protons are at 7.1–7.5 ppm, aliphatic methine and methylene protons are showing between 3 and 4 ppm. In ^13^C NMR 7 carbons were showing in aliphatic region and 11 signals were showing in aromatic region. More de-shielding carbon is phenolic OH located quaternary carbon, remain aromatic signals were shown between 100 and 140 ppm. In aliphatic region O- attached methylene and methine carbon were showing between 55 and 90 ppm, aromatic methylene carbons are at 37.70 ppm. in HSQC experiment confirmed the shifts of methylene and methine carbons. In COSY experiment H24 proton showed connectivity to H19, H23 shows with H20, the H22 proton is with H21 proton, and H28 shows correlation with H25 protons. These connectivity’s are helped to identify the methine and methylene protons. In HMBC experiment, few important ^3^J correlation are observed H1, H3 and H27 protons are showing connectivity to C16 carbon. Also, H7 protons showing ^3^J connectivity to C3, C5 and C9, C13; 2J connectivity to C4, C8 carbons. NMR and mass data confirmed that ethoxy group leaved from the parent and formed phenolic OH on 1,4 disubstituted ring. The structure of the DP-5 has been elucidated and the NMR spectrum portrayed in Figs. 7 and S7. The chemical shifts were in Table 3 and the proposed degradation mechanism was shown in Fig. 5. The FT-IR analysis also confirms the presence of OH group at 3443 cm^−1^ stretching frequency. Stretching frequency at 755 cm^−1^ directs the presence of the C–Cl group. No signal in the region of 1750–1600 cm^−1^ confirms the absence of the keto group in the structure. Central frequencies established the presence of alcohol, Chloride functional groups, and data is tabulated in Table 4. For DP-5, HRMS/MS spectrum and proposed tentative mechanism for protonated fragmentation ions are captured in Figs. 4 and S1.

## Conclusion

The degradation study of Ertugliflozin under stressed conditions was examined following ICH guidelines. The API was subjected to oxidative, acidic, alkaline, neutral, photolytic, and thermolytic degradation conditions. The drug was stable in basic, neutral, thermal, and photolytic conditions, and no degradation products were observed. However, five degradation products were formed in acid and oxidative stress hydrolysis conditions. These degradants are developed because of rearrangement of the ring, which is labile to acid hydrolysis (DP-1, DP-2, DP-3 and DP-4), and DP-5 was formed by removing the ethyl group in the presence of peroxide conditions. All degradation products were separated and characterized entirely using various analytical techniques like NMR (1D and 2D experiments) and HRMS/MS experiments. FT-IR data gave an additional add-on to confirm the structures. All five DPs are novel products and are not reported in any literature. The current study provides the complete structural interpretation of Ertugliflozin and all 5 degradation products using HRMS, FTIR, and 2D-NMR studies. It also reports well developed UHPLC-MS method to separate all the degradation products with good resolution.

## Supplementary Information

Supplementary Figure S1.

Supplementary Figure S2.

Supplementary Figure S3.

Supplementary Figure S4.

Supplementary Figure S5.

Supplementary Figure S6.

Supplementary Figure S7.

Supplementary Legends.

## Data Availability

All data generated or analyzed during this study are included in this published article and its supplementary information files.

## Abbreviations

ERG: Ertugliflozin
API: Active pharmaceutical ingredient
ICH: International council for harmonisation
SGLT2: Sodium Glucose co-transporter 2
UHPLC-MS: Ultra-high performance liquid chromatography-mass spectrometry
FTIR: Fourier transform infrared spectroscopy
HRMS: High resolution mass spectrometry
2-D NMR: Two-dimensional nuclear magnetic resonance spectroscopy
SQD: Single quadrupole detector
PDA: Photo Diode array detector
ESI: Electrospray ionisation
gDQFCOSY: Gradient Double Quantum Filtered Correlated Spectroscopy
gHSQC: Gradient Heteronuclear Single Quantum Coherence spectroscopy
gHMBC: Gradient Heteronuclear Multiple Bond Coherence spectroscopy
