# Supplementary figures and images for: Identification, isolation, and structural characterization of novel forced degradation products of Ertugliflozin using advanced analytical techniques
Source: Sci Rep. 2023 Jun 10;13:9472. doi: 10.1038/s41598-023-36289-9 (PMC10257675; doi:10.1038/s41598-023-36289-9)

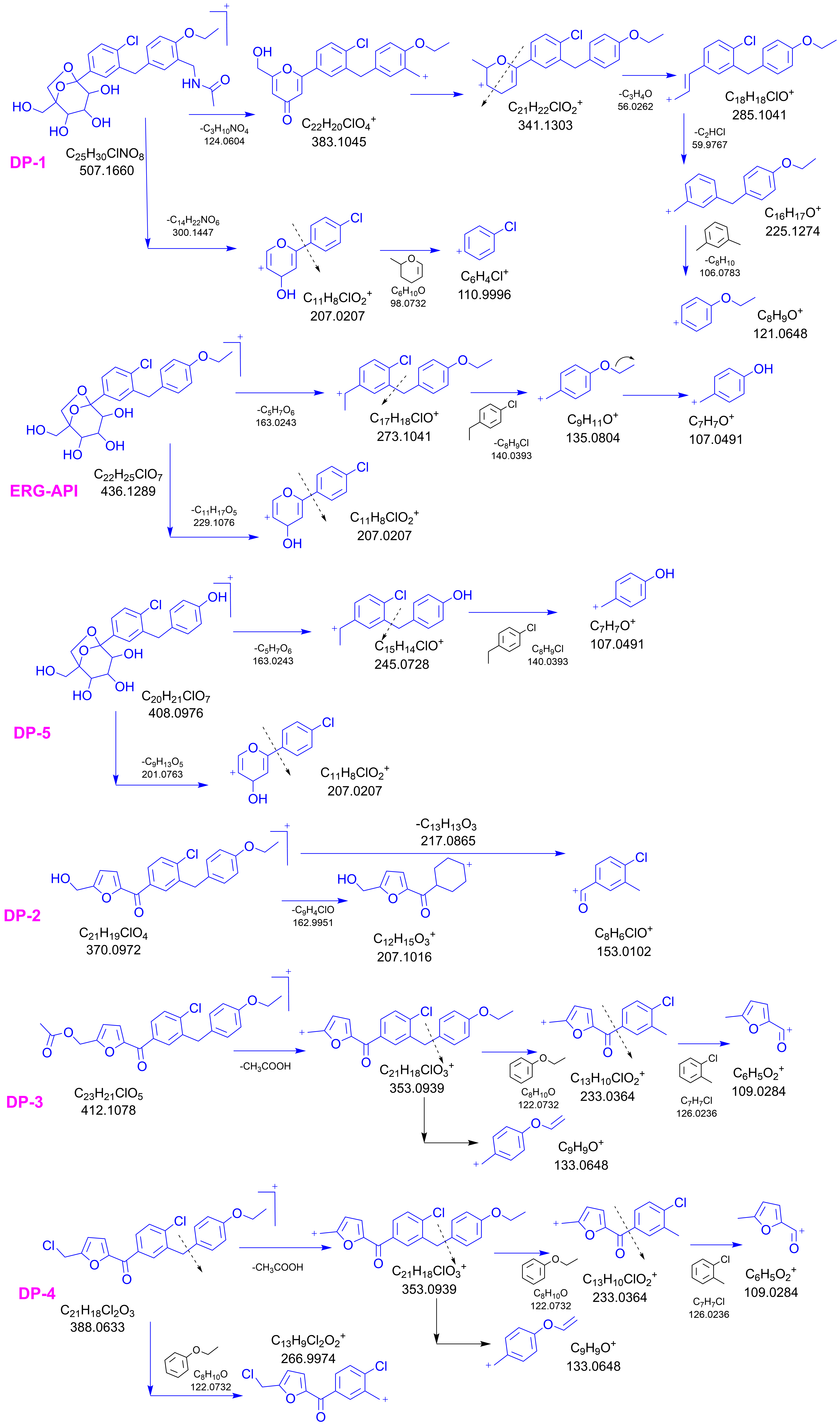

Supplement: Supplementary file 1 — Supplementary Figure S1. [file 41598_2023_36289_MOESM1_ESM.tif]
