# Supplementary material for: Identification, isolation, and structural characterization of novel forced degradation products of Ertugliflozin using advanced analytical techniques
Source: Sci Rep. 2023 Jun 10;13:9472. doi: 10.1038/s41598-023-36289-9 (PMC10257675; doi:10.1038/s41598-023-36289-9)
Supplement: Supplementary file 2 — Supplementary Figure S2. [file 41598_2023_36289_MOESM2_ESM.docx]

**Identification, Isolation, and Structural Characterization of Novel Forced Degradation Products of Ertugliflozin using Advanced Analytical Techniques UPLC-MS, PREP-HPLC, HRMS, FT-IR, and 2D-NMR.**

Suresh Salakolusu^a,b^, Ganapavarapu Veera Raghava Sharma^b*^, Naresh Kumar Katari^c*^, Muralidharan Kaliyaperumal ^a^, Umamaheshwar Puppala^a^, Mahesh Ranga^a^, Sreekantha Babu Jonnalagadda^d^.

**Analytical data for Ertugliflozin API:**

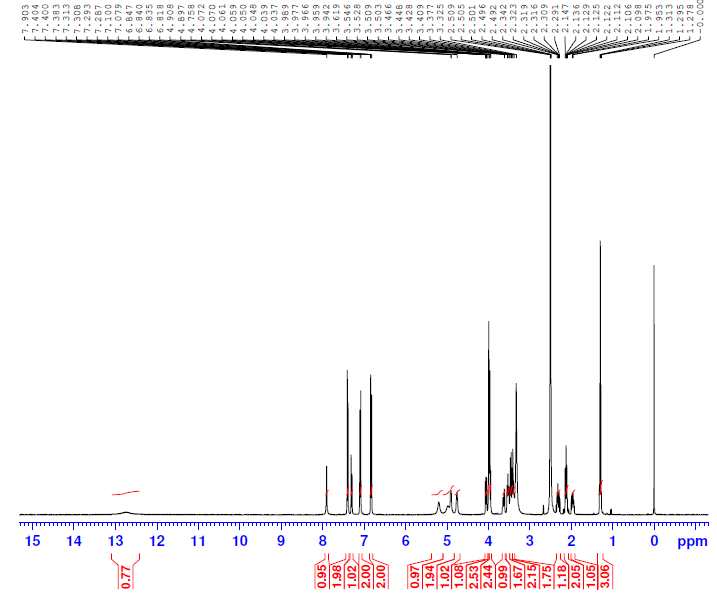


ERG- API ^1^H NMR


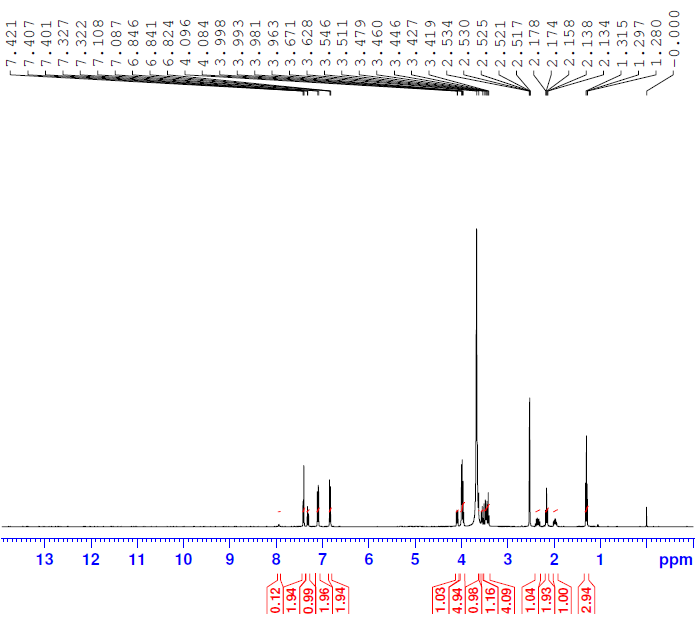


ERG- ^1^H NMR-1-D_2_O


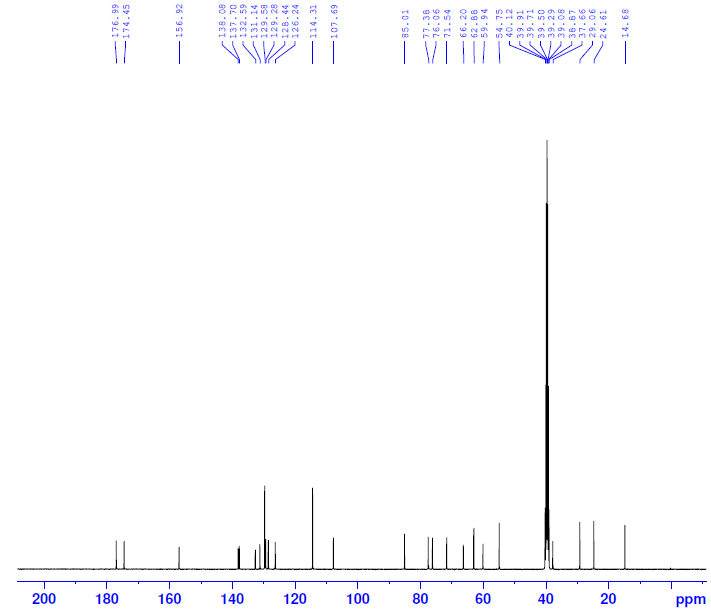


ERG- API ^13^C NMR


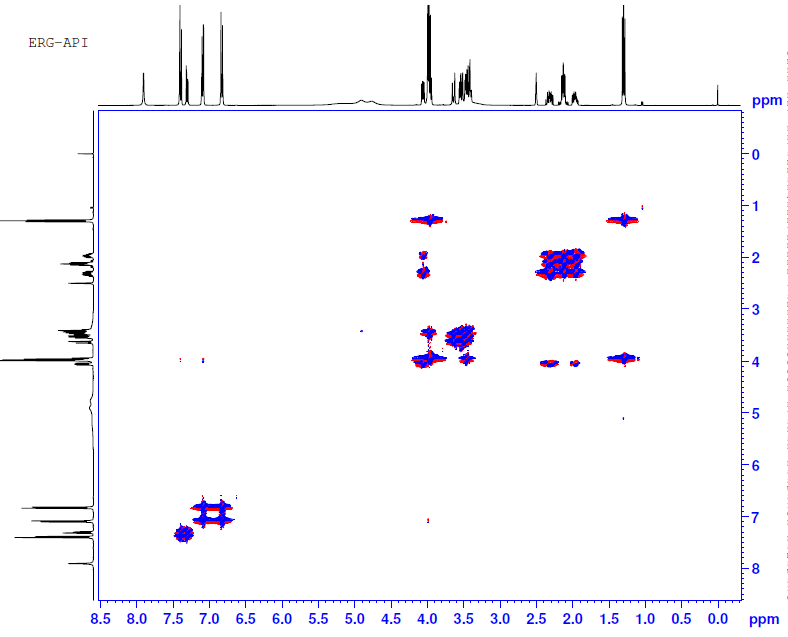


ERG- API COSY NMR


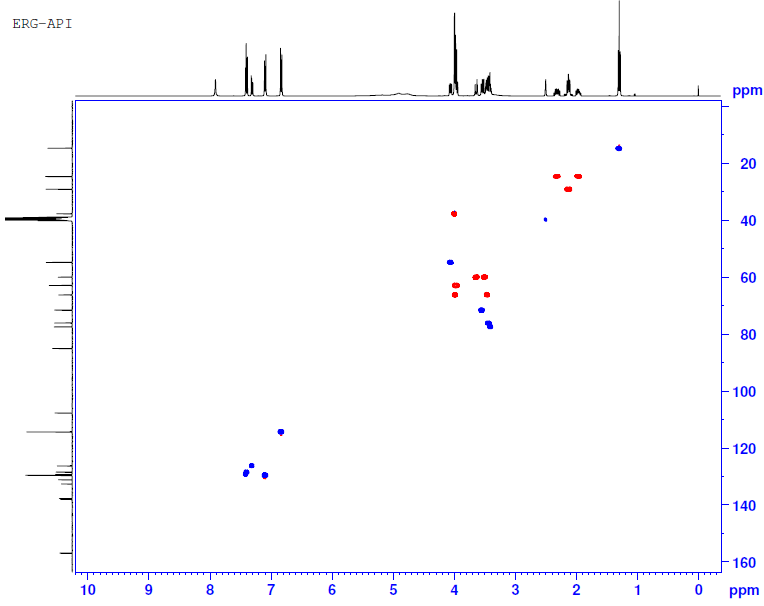


ERG- API HSQC NMR


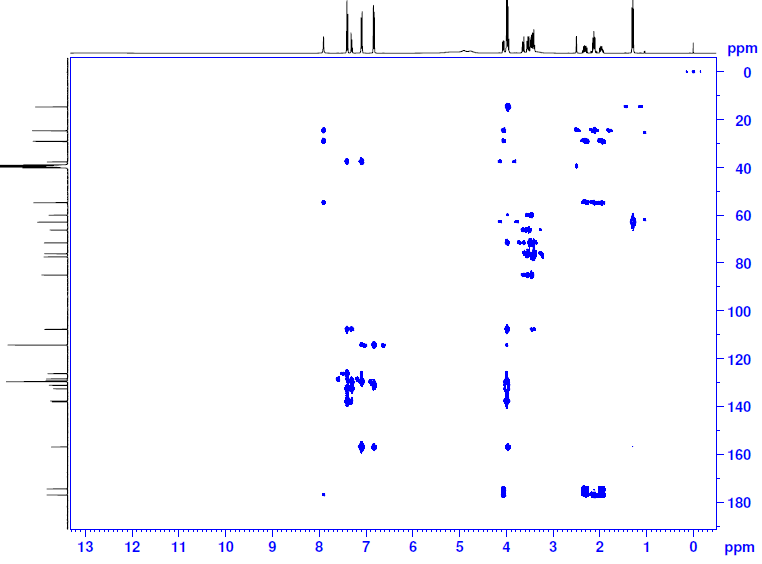


ERG- API HMBC NMR


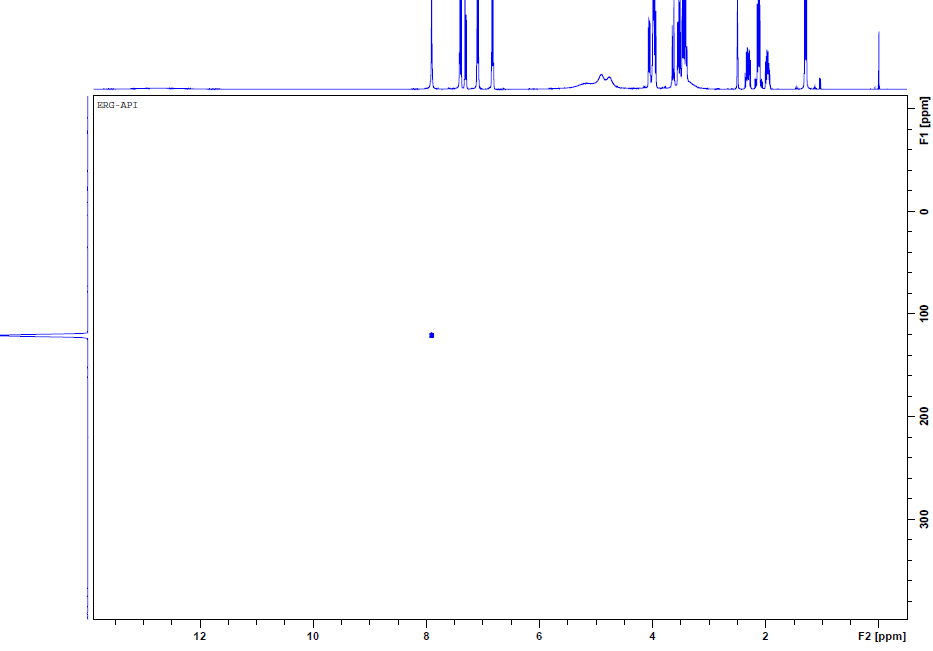
 ERG- API ^15^N HSQC NMR


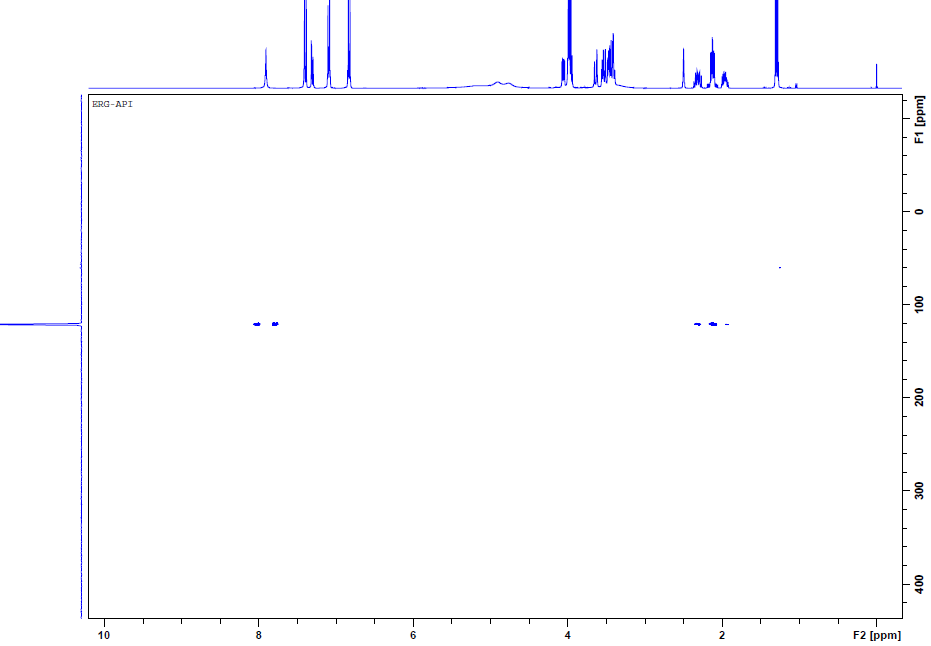


ERG- API ^15^N HMBC NMR


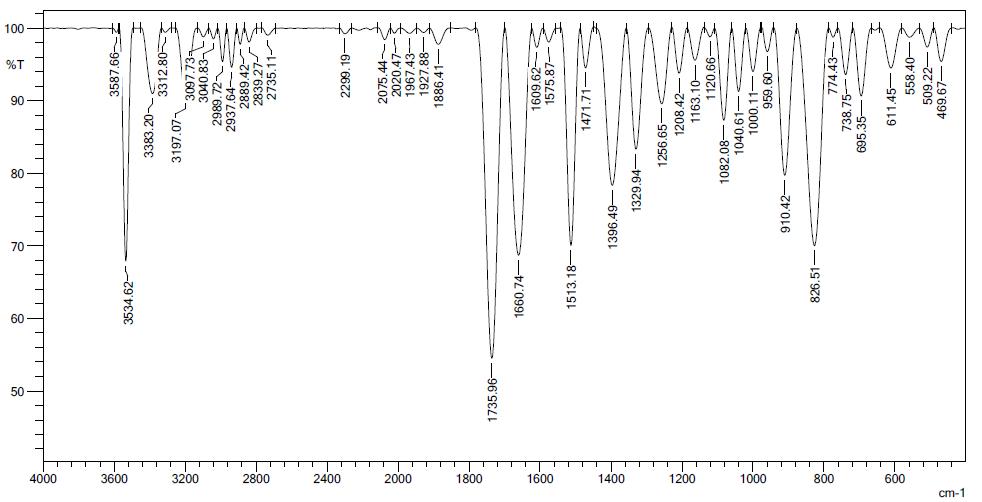


ERG- API -IR

ERG-API-HRMS

ERG-API-HRMS-MS
