# Supplementary material for: Identification, isolation, and structural characterization of novel forced degradation products of Ertugliflozin using advanced analytical techniques
Source: Sci Rep. 2023 Jun 10;13:9472. doi: 10.1038/s41598-023-36289-9 (PMC10257675; doi:10.1038/s41598-023-36289-9)
Supplement: Supplementary file 3 — Supplementary Figure S3. [file 41598_2023_36289_MOESM3_ESM.docx]

**Identification, Isolation, and Structural Characterization of Novel Forced Degradation Products of Ertugliflozin using Advanced Analytical Techniques UPLC-MS, PREP-HPLC, HRMS, FT-IR, and 2D-NMR.**

Suresh Salakolusu^a,b^, Ganapavarapu Veera Raghava Sharma^b*^, Naresh Kumar Katari^c*^, Muralidharan Kaliyaperumal ^a^, Umamaheshwar Puppala^a^, Mahesh Ranga^a^, Sreekantha Babu Jonnalagadda^d^.

**Analytical data for Ertugliflozin degradation product-1:**

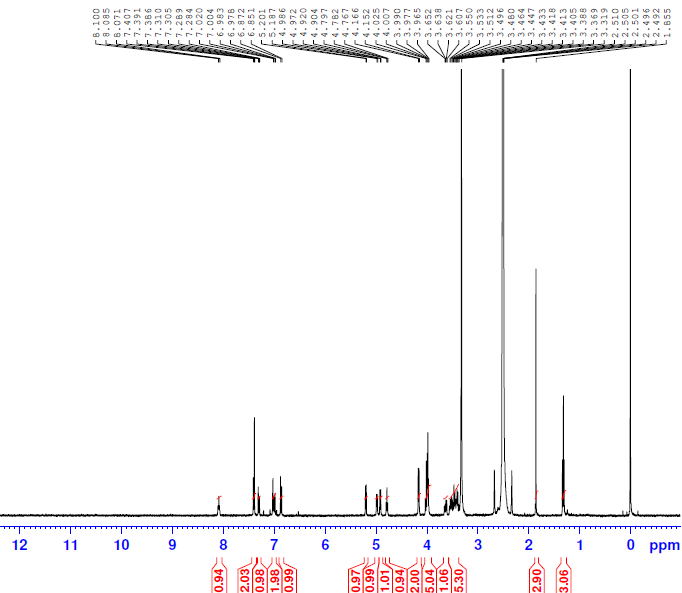


ERG-DP-1- ^1^H NMR


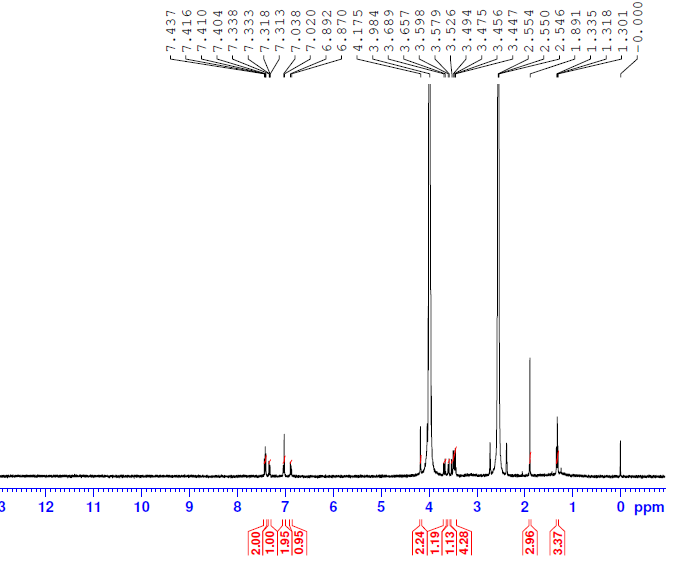
ERG-DP-1- ^1^H NMR-D2O


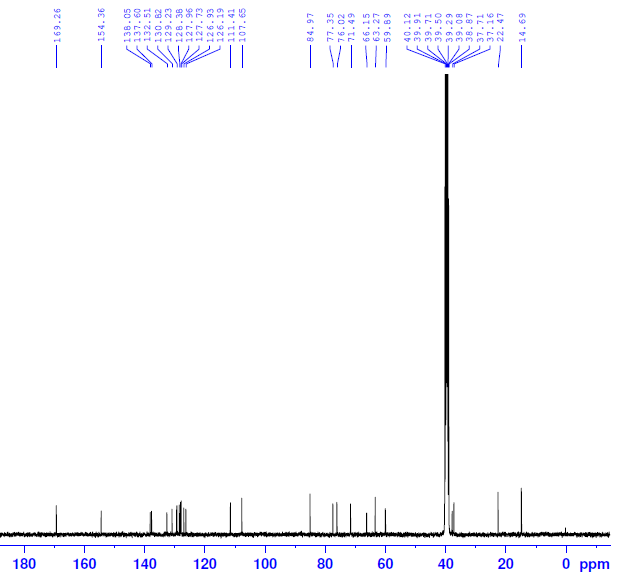


ERG-DP-1- ^13^C NMR


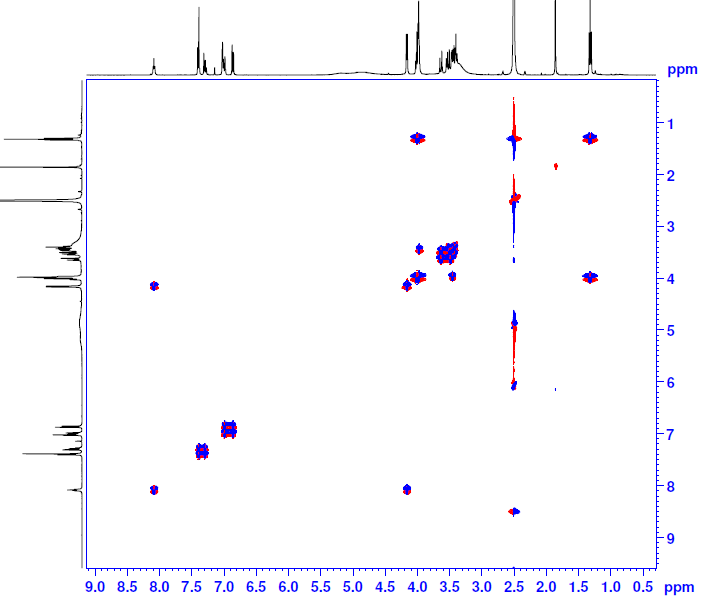


ERG-DP-1- COSY


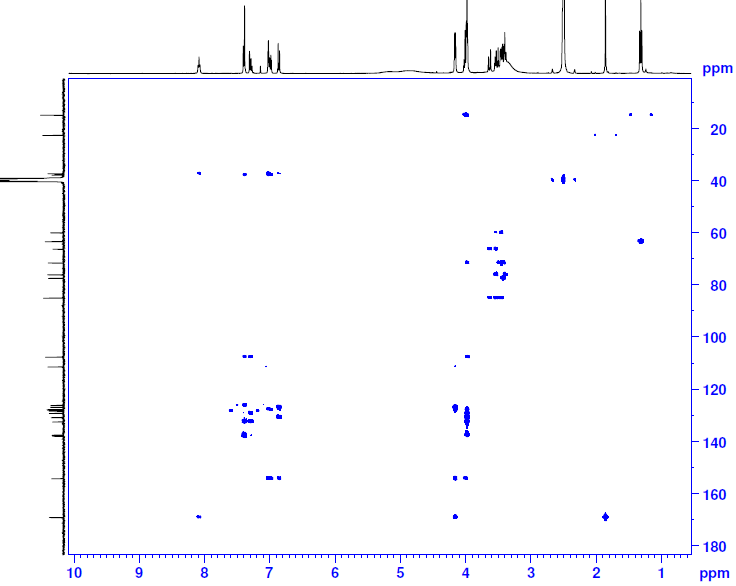


ERG-DP-1-HMBC-2


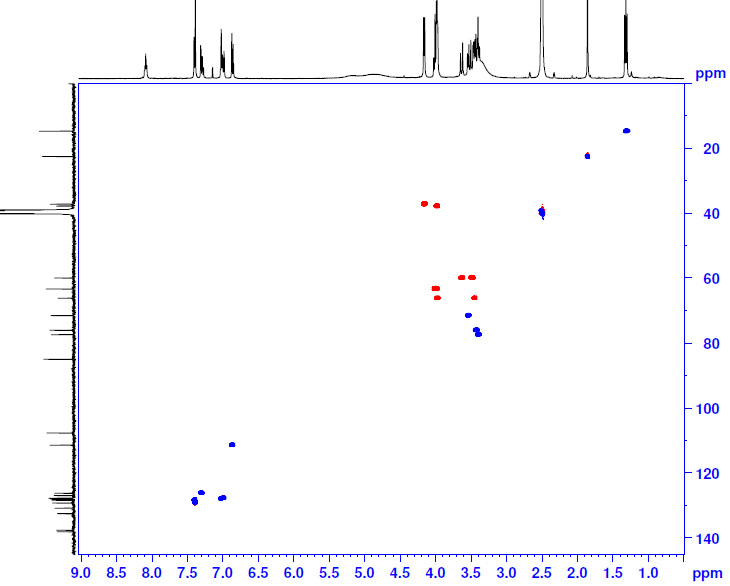


ERG-DP-1- HSQC


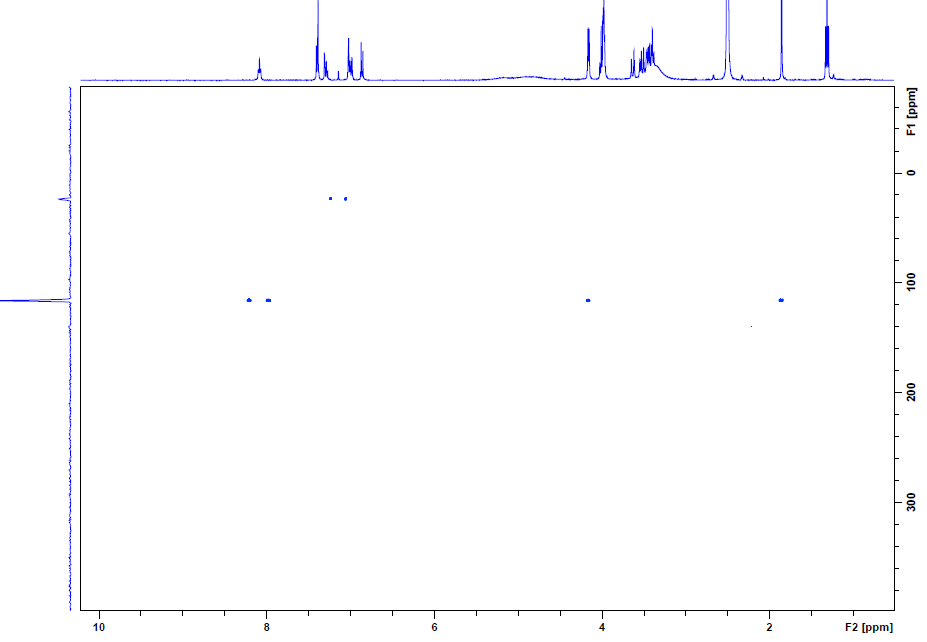


ERG-DP-1- ^15^N HMBC


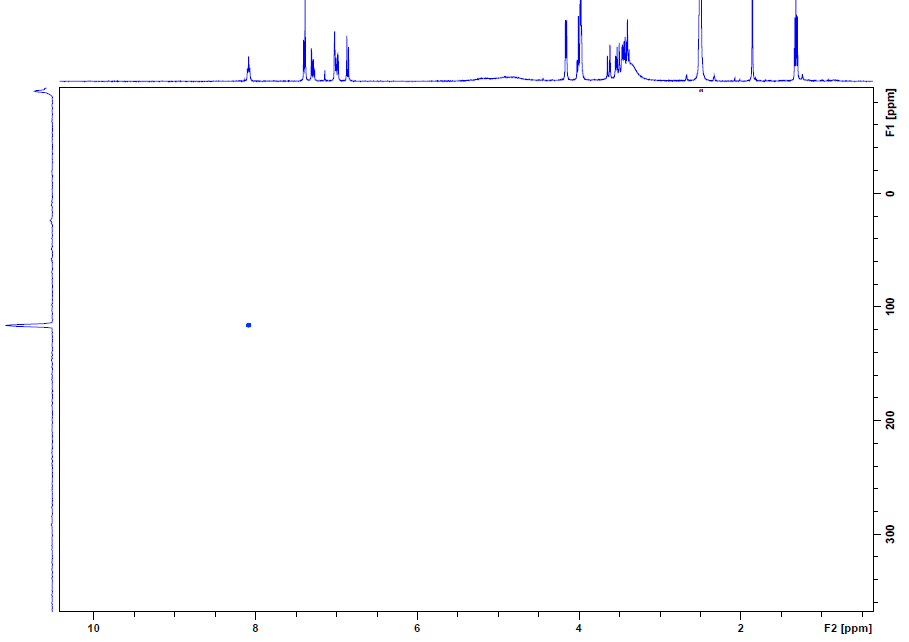


ERG-DP-1-^15^N HSQC-1


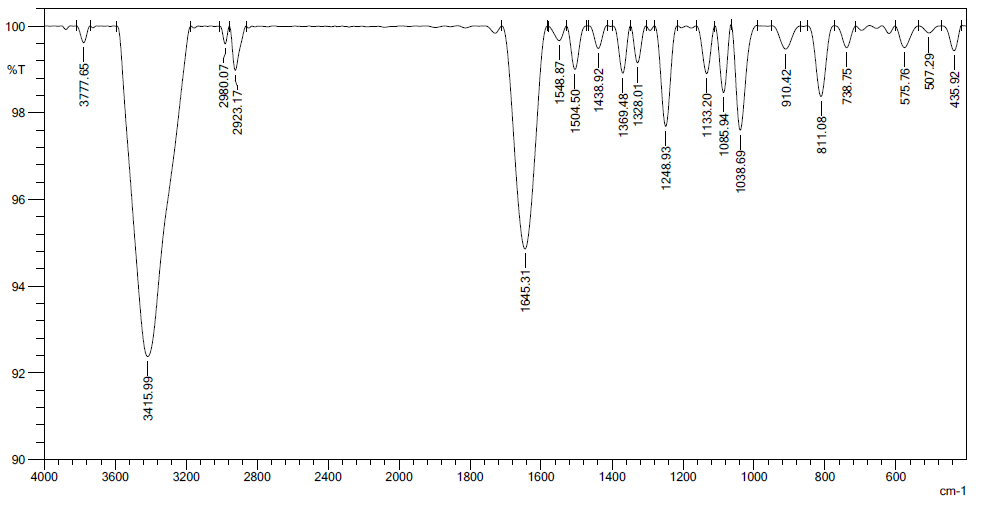


ERG-DP-1- IR

ERG-DP-1- HRMS

ERG-DP-1- HRMS-MS
