# Supplementary material for: Identification, isolation, and structural characterization of novel forced degradation products of Ertugliflozin using advanced analytical techniques
Source: Sci Rep. 2023 Jun 10;13:9472. doi: 10.1038/s41598-023-36289-9 (PMC10257675; doi:10.1038/s41598-023-36289-9)
Supplement: Supplementary file 6 — Supplementary Figure S6. [file 41598_2023_36289_MOESM6_ESM.docx]

**Identification, Isolation, and Structural Characterization of Novel Forced Degradation Products of Ertugliflozin using Advanced Analytical Techniques UPLC-MS, PREP-HPLC, HRMS, FT-IR, and 2D-NMR.**

Suresh Salakolusu^a,b^, Ganapavarapu Veera Raghava Sharma^b*^, Naresh Kumar Katari^c*^, Muralidharan Kaliyaperumal ^a^, Umamaheshwar Puppala^a^, Mahesh Ranga^a^, Sreekantha Babu Jonnalagadda^d^.

**Analytical data for Ertugliflozin degradation product-4:**

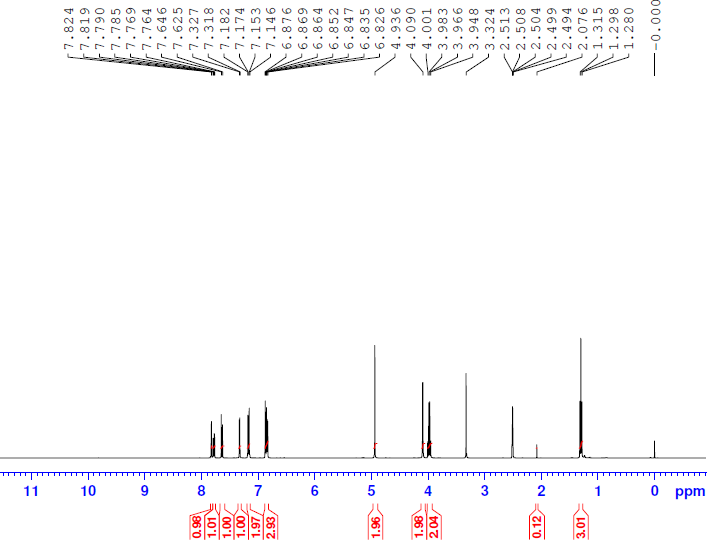
-ERG-DP-04 ^1^H NMR


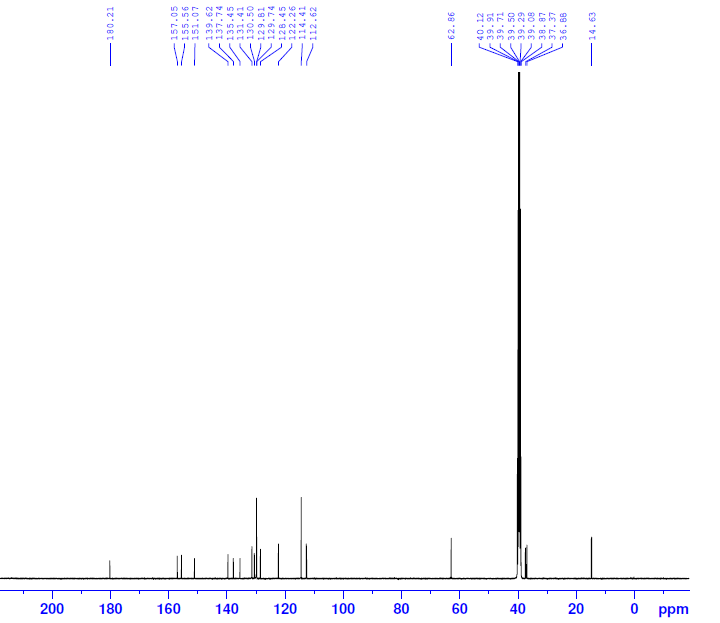


ERG-DP-04 ^13^C NMR


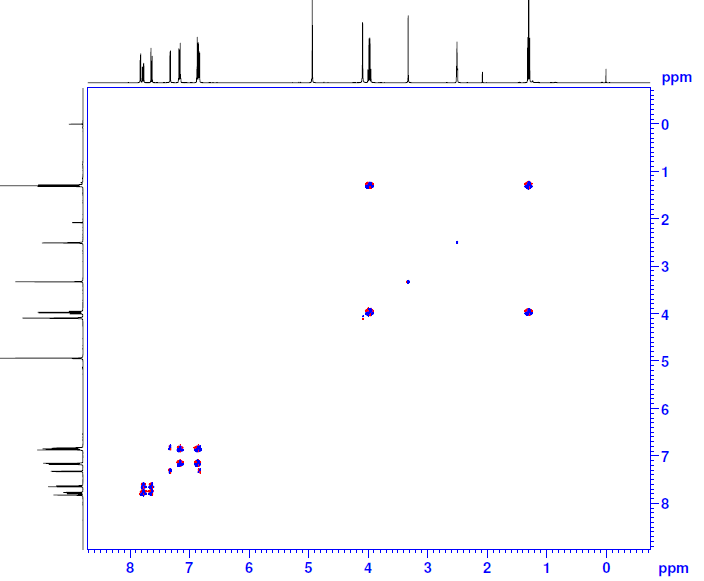


ERG-DP-04 COSY NMR


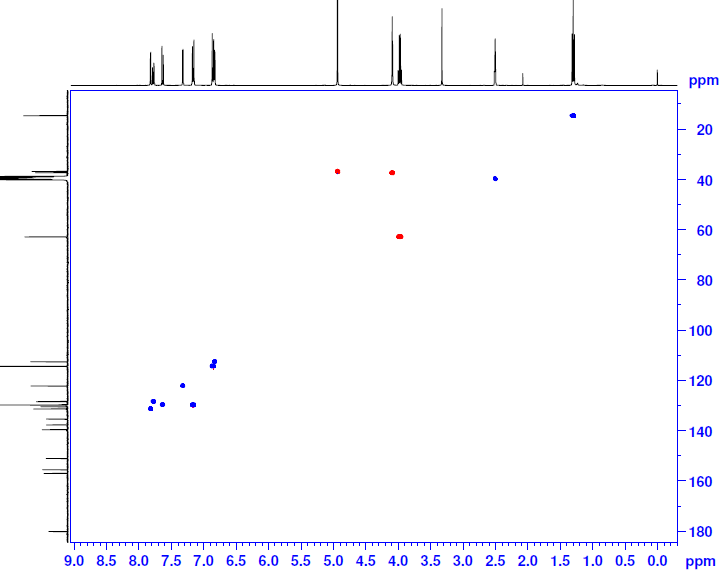


ERG-DP-04 HSQC NMR


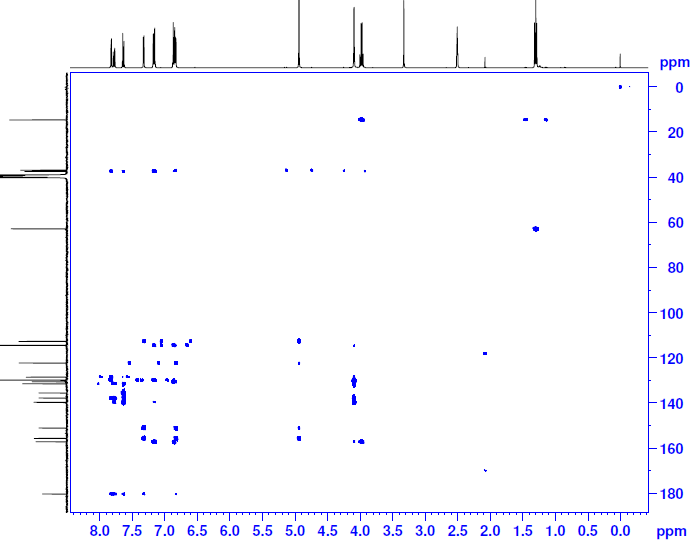


ERG-DP-04 HMBC NMR


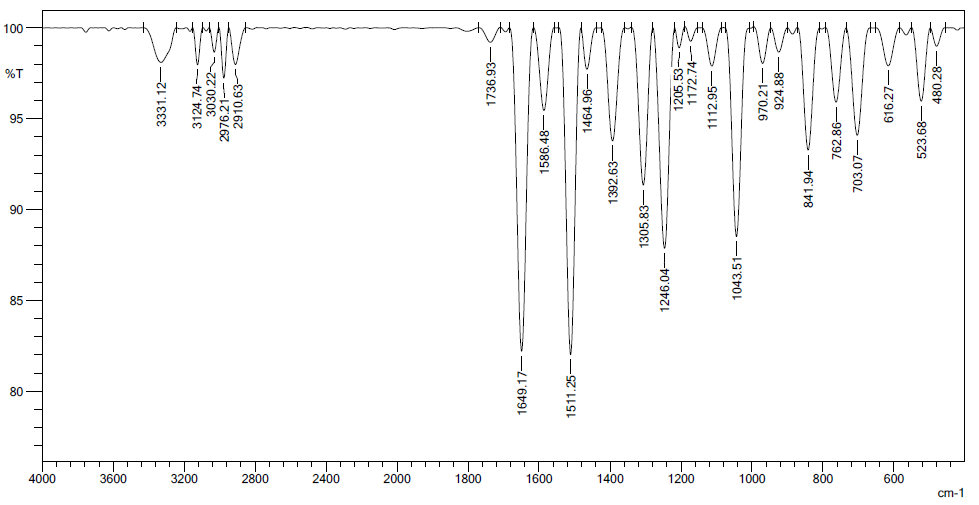


ERG-DP-04-IR

ERG-DP-04-HRMS

ERG-DP-04-HRMS-MS
