# Supplementary material for: Identification, isolation, and structural characterization of novel forced degradation products of Ertugliflozin using advanced analytical techniques
Source: Sci Rep. 2023 Jun 10;13:9472. doi: 10.1038/s41598-023-36289-9 (PMC10257675; doi:10.1038/s41598-023-36289-9)
Supplement: Supplementary file 8 — Supplementary Legends. [file 41598_2023_36289_MOESM8_ESM.docx]

Figure-S1 Proposed structures for the fragmented protenated ions in HRMS-MS

Figure-S2 NMR & IR data for ertugliflozin

Figure-S3 NMR & IR data for degradation product-1

Figure-S4 NMR & IR data for degradation product-2

Figure-S5 NMR & IR data for degradation product-3

Figure-S6 NMR & IR data for degradation product-4

Figure-S7 NMR & IR data for degradation product-5
